# Commonly observed sex differences in direct aggression are absent or reversed in sibling contexts

**DOI:** 10.1093/pnasnexus/pgaf239

**Published:** 2025-08-26

**Authors:** Michael E W Varnum, Amanda P Kirsch, Daniel J Beal, Cari M Pick, Laith Al-Shawaf, Chiara Ambrosio, Maria Teresa Barbato, Oumar Barry, Watcharaporn Boonyasiriwat, Eduard Brandstätter, Suzan Ceylan-Batur, Marco Antonio Correa Varella, Julio Eduardo Cruz, Oana David, Laina Ngom Dieng, Dimitri Dubois, Ana María Fernandez, Silvia Galdi, Oscar Javier Galindo Caballero, Sylvie Graf, Igor Grossmann, David Guzman, Peter Halama, Takeshi Hamamura, Martina Hřebíčková, Ioana Iuga, Lady Javela, Jaewuk Jung, Johannes A Karl, Jinseok P Kim, Michal Kohút, Anthonieta Looman Mafra, Dieynaba Gabrielle Ndiaye, Jiaqing O, Beatriz Perez Sánchez, Eric Roth Unzueta, Muhammad Rizwan, A Timur Sevincer, Eric Skoog, Eunkook M Suh, Daniel Sznycer, Evelina Thunell, Arnaud Tognetti, Ayse K Uskul, Jaroslava Varella Valentova, Yunsuh Nike Wee, Anja Lundkvist Winter, Torin Peter Young, Danilo Zambrano, Anna Ziska, Douglas T Kenrick

**Affiliations:** Department of Psychology, Arizona State University, Tempe, AZ 85282-1104, USA; Department of Psychology, Arizona State University, Tempe, AZ 85282-1104, USA; Department of Management, Virginia Tech, Blacksburg, VA 24061, USA; Office of the Chief Scientist, Environmental Defense Fund, New York, NY 10100, USA; Department of Psychology, Center for Cognitive Archaeology, Lyda Hill Institute for Human Resilience, University of Colorado Colorado Springs, Colorado Springs, CO 80918, USA; Department of Psychology, University of Campania “Luigi Vanvitelli”, Caserta 81100, Italy; School of Psychology, Universidad de Santiago de Chile, Santiago 9170022, Chile; Department of Psychologie, University Cheikh Anta Diop, Dakar 10700, Senegal; The Faculty of Psychology, Chulalongkorn University, Bangkok 10330, Thailand; Institut für Psychologie, Johannes Kepler University Linz, Linz 4040, Austria; Department of Psychology, TOBB University of Economics and Technology, Ankara 06510, Türkiye; Department of Experimental Psychology, Institute of Psychology, University of São Paulo, São Paulo 05508-030, Brazil; Department of Psychology, Universidad de los Andes, Bogotá 111711, Colombia; The International Institute for the Advanced Studies of Psychotherapy and Applied Mental Health, Babes-Bolyai University Cluj-Napoca, Cluj-Napoca 400347, Romania; Department of Psychologie, University Cheikh Anta Diop, Dakar 10700, Senegal; CEE-M, University Montpellier, CNRS, INRAE, Institut Agro, Montpellier 34000, France; School of Psychology, Universidad de Santiago de Chile, Santiago 9170022, Chile; Department of Psychology, University of Campania “Luigi Vanvitelli”, Caserta 81100, Italy; Faculty of Psychology, Universidad Católica de Colombia, Bogotá 110911, Colombia; Institute of Psychology, Czech Academy of Sciences, Prague 11000, Czechia; Department of Psychology, University of Waterloo, Waterloo, Ontario, Canada N2L 3G1; Faculty of Psychology, Fundación Universitaria Konrad Lorenz, Bogotá 110231, Colombia; Institute of Experimental Psychology, Slovak Academy of Sciences, Bratislava 841 04, Slovakia; School of Psychology, Curtin University, Perth 6102, Australia; Institute of Psychology, Czech Academy of Sciences, Prague 11000, Czechia; The International Institute for the Advanced Studies of Psychotherapy and Applied Mental Health, Babes-Bolyai University Cluj-Napoca, Cluj-Napoca 400347, Romania; Department of Psychology, Universidad del Rosario, Bogotá 110311, Colombia; Department of Psychology, Sogang University, Seoul 04107, South Korea; School of Psychology, Victoria University of Wellington, Wellington 6140, New Zealand; Stanford Graduate School of Business, Stanford, CA 94305, USA; Department of Psychology, Yonsei University, Seoul 03722, South Korea; Department of Psychology, University of Trnava, Trnava 918 43, Slovakia; Department of Experimental Psychology, Institute of Psychology, University of São Paulo, São Paulo 05508-030, Brazil; Department of Psychologie, University Cheikh Anta Diop, Dakar 10700, Senegal; Department of Psychology, Aberystwyth University, Aberystwyth SY23 3UX, United Kingdom; Department of Psychology, University of La Frontera, Temuco 4780000, Chile; Department of Psychology, Universidad Católica Boliviana, La Paz 0201-0220, Bolivia; Department of Clinical Psychology, National University of Medical Sciences, Rawalpindi 46000, Pakistan; Institute of Psychology, Leuphana University of Lüneburg, Lüneburg 21335, Germany; Peace Research Institute Oslo, Oslo NO-0134, Norway; Department of Psychology, Yonsei University, Seoul 03722, South Korea; Department of Psychology, Oklahoma State University, Stillwater, OK 74078, USA; Division of Psychology, Department of Clinical Neuroscience, Karolinska Institutet, Stockholm 171 77, Sweden; CEE-M, University Montpellier, CNRS, INRAE, Institut Agro, Montpellier 34000, France; Division of Psychology, Department of Clinical Neuroscience, Karolinska Institutet, Stockholm 171 77, Sweden; School of Psychology, University of Sussex, Brighton and Hove BN1 9RH, United Kingdom; Department of Experimental Psychology, Institute of Psychology, University of São Paulo, São Paulo 05508-030, Brazil; Department of Psychology, Oklahoma State University, Stillwater, OK 74078, USA; Division of Psychology, Department of Clinical Neuroscience, Karolinska Institutet, Stockholm 171 77, Sweden; Department of Psychology, University of Waterloo, Waterloo, Ontario, Canada N2L 3G1; Faculty of Psychology, Fundación Universitaria Konrad Lorenz, Bogotá 110231, Colombia; Fresenius University of Applied Sciences, Idstein 65510, Germany; Department of Psychology, Arizona State University, Tempe, AZ 85282-1104, USA

**Keywords:** aggression, sex differences, siblings, universal

## Abstract

Decades of research support the generalization that human males tend to be more aggressive than females. However, most of that research has examined aggression between unrelated individuals. Data drawn from 24 societies around the globe (*n* = 4,013) indicate that this generalization does not hold in the context of sibling relationships. In retrospective self-reports, females report being at least as aggressive as males toward their siblings, often more so. This holds for direct as well as indirect aggression, and for aggression between adult siblings as well as aggression that occurred during childhood. Consistent with prior research on sex differences, males reported engaging in more direct aggression toward nonkin than did females in the majority of societies. The results suggest that the dynamics of aggression within the family are different from those outside of it, and ultimately that understanding the role of sex in aggressive tendencies depends on context and target.

Significance Statement A large body of prior work finds that human males tend to be more prone to engage in direct forms of aggression, such as hitting or yelling, than human females. In the present work, we test whether such sex differences are found in the context of sibling relationships. We find that across 24 diverse societies, females and males tend to be equally likely to engage in direct (as well as indirect) forms of aggression toward siblings, whereas in the majority of societies, males report more frequent direct aggression toward nonkin than females. These findings suggest we should take a more nuanced view of how sex relates to aggression.

## Introduction

Are sex differences in aggression universal? A large body of evidence suggests that human males are more prone to aggression than human females (1–3). The vast majority of homicides are committed by males across societies and time periods (2), for example, male high-school students are nearly twice as likely as female high-school students to report having been in a fight in the past 12 months (4). Scholars have argued that these differences may stem from a range of sources, including evolved differences in psychology (1, 2), childhood socialization practices (5), and societal roles and norms (6, 7).

However, might such differences in aggressive tendencies depend on context? And in particular, might the context of sibling relations look different from how men and women behave toward nonkin? Most of the research indicating higher male aggression has examined violence outside of the family. The same sex differences in aggression observed among nonkin might be expected between siblings to the extent that the family is where people learn and practice typical sex roles (in line with socialization theory, 6, 8). Such differences could also arise as byproducts of proximate mechanisms such as higher testosterone levels in boys (9, 10). Further, prior work suggests that in sibling interactions from as early as age 4, parents are more tolerant of various forms of aggression toward siblings committed by boys than they are of such behavior among girls (11). Thus, one might expect that the typical sex differences observed outside the family might also be apparent when the target of aggression is a sibling. And in fact, some studies have found that boys report more sibling conflict than girls (12), and that violent conflict is more common among brothers than among sisters (13, 14).

Yet, one recent study found that aggression among sisters was in fact more common than aggression among brothers (15). And another set of three studies suggests that in interactions between siblings, females are at least as likely to engage in aggression as males are (16). In this set of studies, this pattern was observed not only when recalling childhood behavior but also in adult interactions between siblings (16). Furthermore, the comparatively high level of aggression by sisters was found not only in reports of aggression committed against siblings but also aggression received by siblings (16).

Of note, this pattern of similarly high aggression toward siblings among the sexes has so far only been observed (to our knowledge) in one cultural context, the United States. Given the wide range of ways in which behavior differs across human societies (17), it is important to understand whether these patterns in aggressive behavior are confined to WEIRD (Western, Educated, Industrialized, Rich, and Democratic) cultures or whether they hold across diverse cultural contexts. Cultural norms and societal structures can greatly influence family dynamics, potentially shaping patterns of sibling aggression in unique ways. Further, although some psychological and behavioral differences between the sexes appear to be largely universal, such as differences in mating preferences (18, 19), sociosexuality (20), and levels of self-esteem and confidence (21), others are more culturally variable. Of relevance to the present work, prior research has found that the magnitude of sex differences in preferences for risk taking, patience, and various forms of social exchange varies across societies (22), as does the magnitude of sex differences in negative emotionality (23) and the magnitude and, to some extent, the direction of sex differences in Dark Triad personality traits (24). Eagly and Wood (6) found that sex differences in preferences for mates high in earning capacity or housekeeping skills were more pronounced in societies with greater gender inequality, which they interpreted as supporting the importance of traditional gender-based social roles. Alternatively, other studies have found that sex differences in other psychological and behavioral tendencies appear to be larger in societies that are wealthier or where general levels of gender equality are higher, a phenomenon known as the gender equality paradox (25).

In the present work, we aim to test whether the absence of such sex differences is observed across a wide range of human societies, including samples from wealthy and poor societies, western and nonwestern societies, and every inhabited continent. We also explored whether any differences in aggression were related to societal indices of economic development and gender inequality.

## Methods

To test the cross-cultural generalizability of initial findings on sibling aggression, we collected data on a variety of aggressive behaviors (both direct and indirect) toward siblings, friends, and acquaintances from participants in 24 countries: Australia, Austria, Bolivia, Brazil, Canada, Chile, Columbia, Czechia, France, Germany, Italy, Lebanon, New Zealand, Pakistan, Romania, Senegal, Slovakia, South Korea, Spain, Sweden, Thailand, Türkiye, the United Kingdom, and the United States (see Table S1 for detailed materials and participant demographics).

Participants from each country were sampled through online panels, university subject pools, or community samples. Overall, we obtained usable data from 4,136 individuals, with a median of 150 individuals per country (*M* = 172.33, SD = 80.09). The sample skewed toward female respondents (71% female, 29% male), who, on average, were in young adulthood (*M* = 26.48, SD = 8.04) and had two to three siblings (*M* = 2.32, SD = 1.83). A more detailed breakdown of each country's sample characteristics is provided in the Supplementary material (Appendix A and Tables S1 and S2).

The study protocol was reviewed by the Arizona State University Human Subjects Institutional Review Board, which ruled the study exempt, approval #00012216. Informed consent was obtained from all participants.

Participants accessed the study either online through Qualtrics or with pen and paper, depending on the resources of the university, and provided informed consent. They were first asked if they had at least one full biological sibling, and those with no biological siblings did not participate in the study. Some surveys were translated into languages relevant to each country by the researchers at the institutions outside of Arizona State University.

Participants were then asked to separately report events that had occurred when they were children (16 years or younger) and as adults (18 years or older), in counterbalanced order. For each time period, participants were asked (in randomized order) how often they had aggressed toward each of the following targets: a sister, brother, female friend, male friend, female acquaintance, and male acquaintance (1 = *never*, 2 = *once*, 3 = *several times*, 4 = *many times*; see Supplementary material for descriptive statistics). Given that individuals do not likely keep precise tallies of instances of aggressive behavior over a span of years, we opted to measure frequency of aggression in this fashion. Potential limitations of this approach are addressed in the Discussion section. Participants indicated if they did not have a sister, brother, male friend, or female friend in both childhood and adulthood and only answered questions about relevant targets.

Questions were asked (in randomized order) about two types of direct aggression (hitting/slapping, yelling) and four types of reputational aggression (sharing harmful gossip about a target with a family member, sharing harmful gossip about a target with nonrelatives, reporting a target's behavior to an authority figure inside the family, and reporting a target's behavior to an authority figure outside the family). Participants were asked about aggression toward each type of target both during childhood and during adulthood. For example, a participant might be randomly assigned to first view:

“For the following questions, imagine behaviors you did ONLY during your childhood or early adolescence (up until the age of 16).”

“Have you ever hit/slapped **a sister**?”

“Have you ever hit/slapped **a brother**?”

“Have you ever hit/slapped **a male friend**?”

Then participants would answer questions about the different types of aggression toward both male and female siblings, friends, and acquaintances (in randomized order, as were the specific questions within each set). We also gathered data on additional variables for exploratory purposes, as well as demographic information (see Supplementary material for full details).

The key question was whether earlier results suggesting comparable levels of aggressive behavior by American women toward siblings would be replicated in a range of cultural contexts.

## Results

### Sex differences observed across countries

**Fig. 1.**
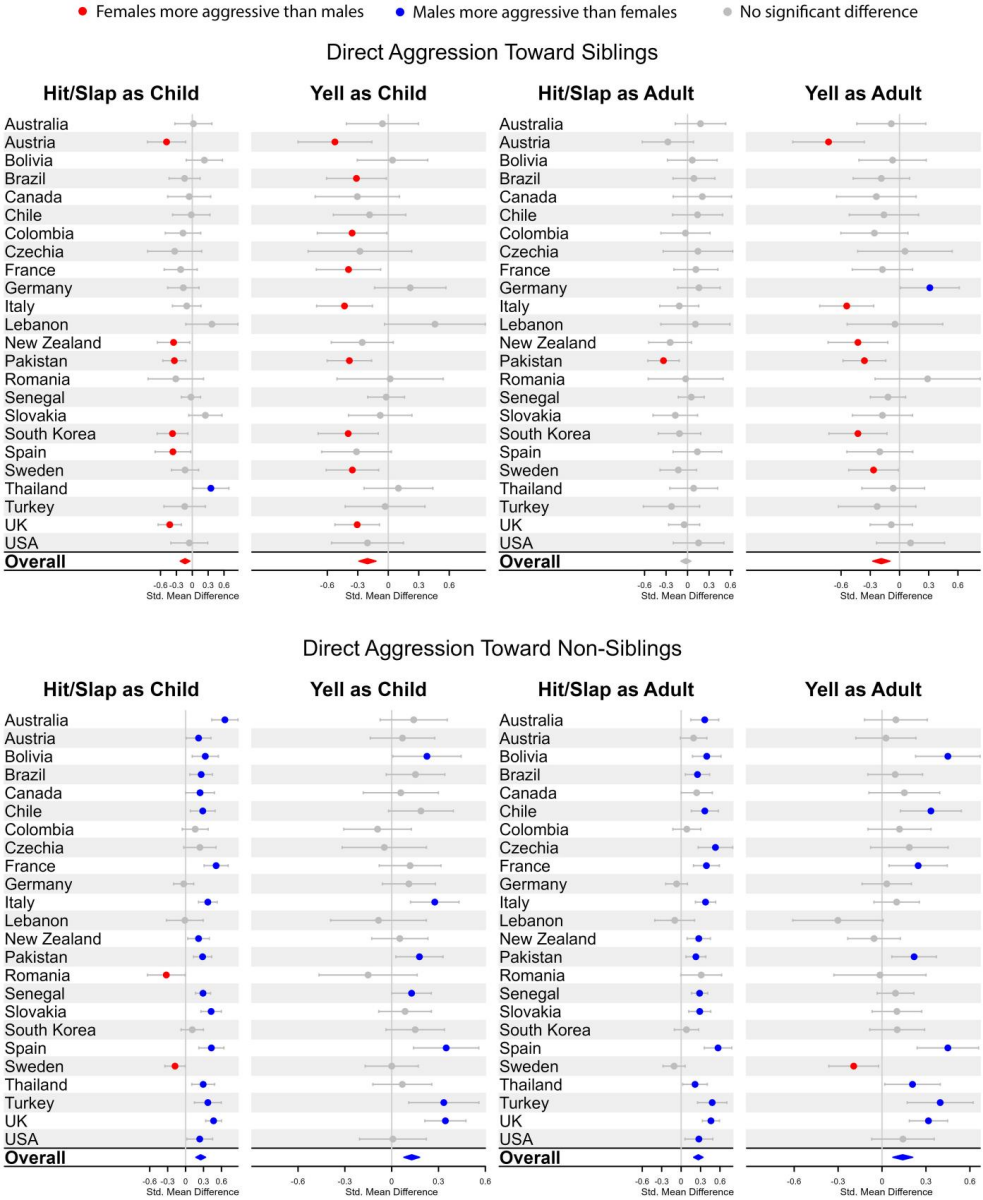
Comparison of female and male direct aggression toward siblings and nonsiblings as both children and adults.

We examined a series of mixed-effects regression models predicting the two direct aggression variables (i.e. hit/slap and yell) based on target of aggression^a^ and sex of participant (for detailed results, see Supplementary material). We first examined these models for direct aggressive acts (i.e. hit/slap and yell) during childhood (i.e. when sibling aggression might be more expected) and then repeated them for aggressive acts during adulthood (i.e. when sibling aggression might perhaps be less likely to occur).^b^ The top left panel of Fig. 1 displays these sex differences for direct aggression toward siblings during childhood. As can be seen in the first two columns, the overall average sex difference across countries indicated that females commit significantly higher levels of direct aggression (i.e. hit/slap and yell) toward siblings when compared with males (for hit/slap, *d* = −0.14, 95% CI = −0.23, −0.04; for yell, *d* = −0.20, 95% CI = −0.29, −0.12). For hit/slap, six countries exhibited significant sex differences, indicating greater direct aggression from females than males toward siblings, with only one significant reversal of this pattern (for Thailand); for yelling, nine countries exhibited significant sex differences, indicating greater direct aggression from females than males, with no countries exhibiting a significant reversal of this pattern.

The top right panel of Fig. 1 displays these effects for direct aggression as an adult and is largely consistent with aggression during childhood, though with slightly weaker effects.

Specifically, the overall sex difference for hitting/slapping siblings during adulthood was nonsignificant (*d* = −0.02, 95% CI = −0.09, 0.05), with only one country exhibiting a significant effect (i.e. Pakistan, in which females were significantly more aggressive than males). For yelling, the overall difference was significant (*d* = −0.19, 95% CI = −0.28, −0.10), indicating greater female than male direct aggression toward siblings. Six countries exhibited significantly greater female than male direct aggression, with one significant reversal of the pattern (for Germany). In sum, these data indicate a pattern of direct aggression toward siblings in which females are slightly more aggressive toward their siblings than are males—a result that stands in marked contrast to the typical pattern of greater male direct aggression.

To ensure that our results were not simply the result of anomalously greater female aggression toward all targets, we examined sex differences in direct aggression toward nonsiblings in the same manner as above. The results exhibited a strikingly different pattern and one that is entirely consistent with past research on sex differences in aggression. As can be seen in the bottom panels of Fig. 1, direct aggression during both childhood and adulthood yielded significant effects for both hit/slap and yell, indicating greater male than female aggression toward nonsibling targets (for hit/slap as child, *d* = 0.25, 95% CI = 0.17, 0.33; for yell as child, *d* = 0.13, 95% CI = 0.08, 0.18; for hit/slap as adult, *d* = 0.27, 95% CI = 0.19, 0.34; for yell as adult, *d* = 0.14, 95% CI = 0.07, 0.21). For hit/slap, the majority of countries exhibited significant effects, indicating greater direct aggression toward nonsiblings from males, and only two countries exhibited a significant reversal (Romania and Sweden for hit/slap during childhood).

**Fig. 2.**
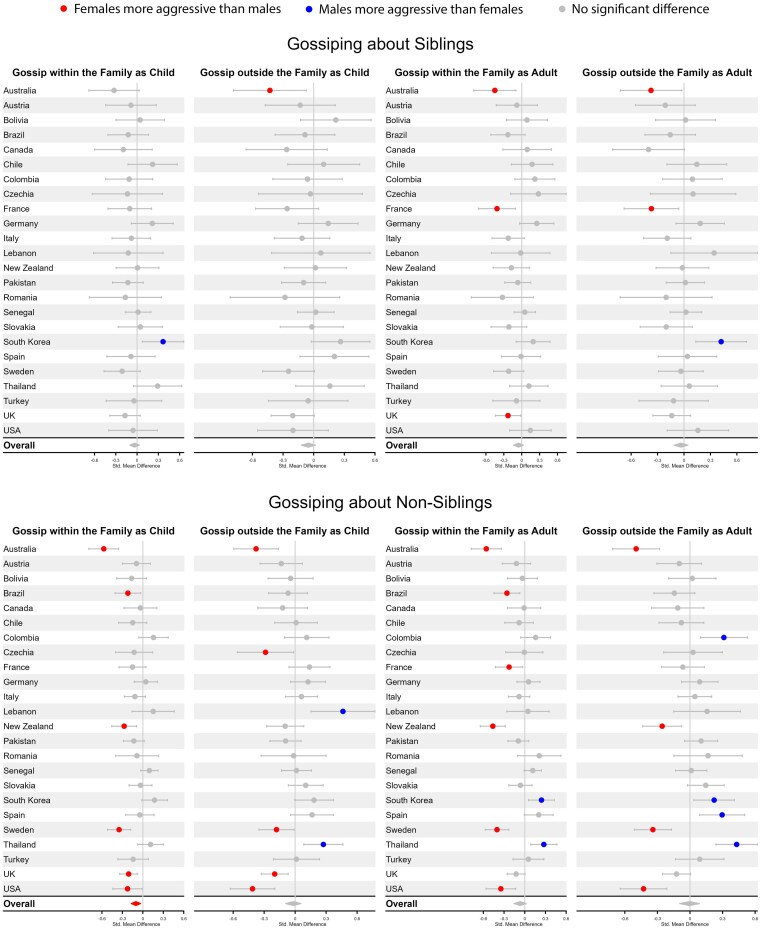
Comparison of female and male gossiping about siblings and nonsiblings as both children and adults.

**Fig. 3.**
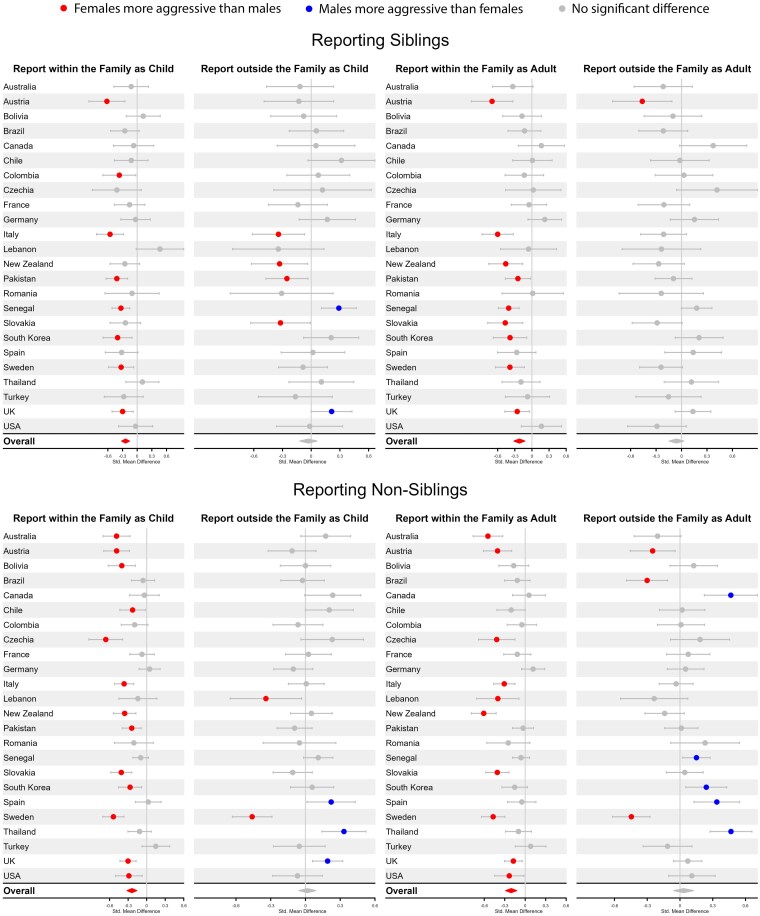
Comparison of female and male reporting siblings and nonsiblings to authority figures as both children and adults.

Previous research indicates that sex differences in indirect aggression are often near zero (26). Our results for indirect aggression—assessed here as gossiping and reporting and displayed in Figs. 2 and 3—exhibited a more nuanced pattern of results. For both gossiping and reporting, we included an additional variable of whether the gossiping or reporting occurred within or outside of the family (see Supplementary material for details). For gossiping, this factor made little difference, as only one small sex difference was detected overall across countries for gossip about nonsiblings to family members as children (*d* = −0.10, 95% CI = −0.17, −0.03). No other significant overall sex differences were found for gossiping. For reporting, however, an interesting pattern emerged in which females, on average, across countries, were more likely to report both siblings (as child, *d* = −0.24, 95% CI = −0.32, −0.16; as adult, *d* = −0.22, 95% CI = −0.32, −0.13) and nonsiblings (as child, *d* = −0.24, 95% CI = −0.32, −0.16; as adult, *d* = −0.21, 95% CI = −0.29, −0.13) to other members of the family, but no significant overall sex differences were observed for reporting siblings or nonsiblings outside the family.

### Variation in sex differences across countries

We also examined the extent to which the effects involving sibling aggression varied across the 24 countries. The first approach we took was to examine the random effect variances for the sex of participant effects for each type of aggression (hit/slap, yell, gossip within family, gossip outside family, report within family, report outside family) and age category (childhood, adulthood). As our models relied on Bayesian estimation, we examined differences in the Deviance Information Criterion (DIC; (27)) to compare models in which the sex of participant effect had either a freely estimated random effect variance or had this parameter fixed to zero (see Supplementary material for details on these comparisons). Importantly, across all of the model comparisons for direct aggression (i.e. hit/slap and yell as adults or as children), results suggested either no notable differences in model fit (i.e. for only hit/slap as a child) or favored the model with the variance parameter fixed to zero (i.e. relying on ΔDIC > ±7; (28)), supporting the idea that sex differences did not vary substantially across countries when considering direct aggression against siblings. For indirect forms of aggression between siblings, a similar pattern of little country-level variance was found for reporting to an authority (for reporting to someone within or outside of the family, either as an adult or as a child), as was the case for gossiping as an adult. Interestingly, however, models that included country-level variance for sex differences were favored when considering gossiping as a child (for both gossiping within or outside the family).

Although this analysis suggests that the sex differences we observed, particularly for direct aggression, are generally shared across the 24 countries examined, this approach does not tell us about whether the reversal of sex differences we found for direct aggression between sibling and nonsibling targets is a pattern that is consistent across countries. We therefore additionally examined the country-level variances for the interactions of sex of participant by family status of target (sibling, nonsibling) for both forms of direct aggression (hit/slap, yell) and both ages (childhood, adulthood). These results offered more nuanced results, with support for no country-level variance for hitting as a child, support for including country-level variance for hitting as an adult, and negligible differences between models with or without country-level variance for yelling, either as a child or as an adult.

Because there was some indication of small amounts of country-level variation in these interaction effects, we next examined whether this variation might be due to substantive cultural or economic variations. Specifically, we examined whether the country-level variance in the four interaction effects (i.e. sex of participant × target relatedness for two direct aggressive behaviors and two ages) might be due to any of three variables: gross domestic product, global gender gap, or cultural distances from the United States (the one country in which this pattern of effects has already been demonstrated; 16) for the 24 countries examined. This allowed us to test whether patterns observed might be unique to WEIRD societies or linked to broader norms about gender equality and gender roles. These country-level variables were merged with the current data from the EcoCultural Dataset (29). To examine the possibility that these factors serve as alternative explanations for the interaction patterns we observed for direct aggression, we added this set of three country-level variables as predictors of the variance in the interaction effects to the models described above. None of these variables emerged as a significant predictor for the country-level variance in the four interaction effects (median *z*-test value was 0.21 and ranged from 0.01 to 1.78; median *P*-value was 0.84 and ranged from 0.08 to 0.99), suggesting that neither economic development nor broader social gender norms nor peculiarities of the US culture likely explain the patterns observed (see Tables S2–S5 for additional results and methodological details).

## Discussion

Data from 24 samples drawn from societies around the globe indicated that females engage in at least as much aggression toward their siblings as males do. This was true not only for indirect forms of aggression, which prior work has suggested may be comparable in general among males and females (1), but also for direct forms of aggression, including mild forms of physical violence. This pattern held in wealthier and poorer countries and in western and nonwestern cultures, suggesting that it may well be universal. Further, females reported being at least as aggressive toward siblings as males, not only when asked to report incidents of aggression during childhood but also when reporting on aggression during interactions in adulthood. Thus, it is unlikely our findings simply reflect greater opportunity for conflict among siblings versus among nonrelatives during childhood. The fact that patterns were similar in adulthood also suggests that socialization of gender norms does not likely explain our findings.

Further, despite societies differing markedly in levels of gender equality, we found that in nearly all societies, and for nearly all types of aggressive behaviors reported, males did not engage in more frequent aggression toward siblings than did females, with very few exceptions. Social role theory posits that differences between men and women in behavior are largely driven by broader societal norms. Hence, one might expect men and women to look more similar in societies in which norms, policies, and practices encourage more equality. However, that was not the case. Further, the patterns of sibling aggression by sex were not correlated with broader societal indicators of gender equality. This suggests that the patterns observed here are unlikely to be explained by broader social norms regarding gender roles. Although the finding that males reported greater direct aggression toward nonkin than females is consistent with social role theory (as well as evolutionary accounts), the finding that females are at least as aggressive toward siblings as males is the opposite of what that theory would predict. The findings regarding aggression within the family are inconsistent not only with predictions of social role theory, of course, but also with evolutionary theories that have been advanced to explain the reliably higher levels of aggression among males outside the family.

There were also no systematic relationships between markers of economic development or broader gender equality and the magnitude of sex differences in aggressive behavior. This suggests that at least for aggressive tendencies there is no evidence consistent with a “gender equality paradox.” That said, our sample size of 24 countries may have been underpowered to detect such correlations.

Why might well-established sex differences in direct aggression outside the family (which were replicated in the present work) be absent or reversed in the context of sibling interactions? Aggression outside the family has been linked to the differential importance of competing for status, which is more connected to mating payoffs for males than for females (2, 3). Outside the family, then, male and female aggression is linked to very different costs and benefits. Within the family, however, males and females are competing for the same payoffs—their share of familial resources. Furthermore, inclusive fitness considerations may reduce the relative danger of aggression in conflicts among siblings, making severe retaliation less likely. Although the types of aggressive behavior that are the focus of this study are more common in sibling dyads, more severe forms of aggression are rarer among close kin than nonkin (2). The potential costs of engaging in physical aggression outside the family are generally higher for females than males, given sexual dimorphism in our species in terms of strength and size. But inside the family, it may be that the “bumpers” that inclusive fitness places on the severity of aggression reduce potential costs of such behavior for females to the point where potential gains in such conflicts outweigh those costs. It is nevertheless still puzzling why females would, in some cases at least, as the current evidence would suggest—especially when direct aggression was involved—be more aggressive toward their siblings than males, if the same inclusive fitness “bumpers” would likely apply to both in an equal manner. One possible explanation is that males were already likely to be physically stronger than females even during childhood (30), and so an act of direct aggression could, by then, be more likely to bring about more physical harm than if the aggressive act was committed by a female on her sibling in childhood. If humans have generally evolved to be conscious about inclusive fitness, it would then make sense that males would be more restrained than their female siblings (and possibly more restrained on certain occasions when physical strength differences were more pronounced) when directly aggressing toward their sibling (especially if the target is one of a different sex).

A related, more proximate, possibility is that parents may be more likely to reprimand sons than daughters for hitting their siblings. There is evidence that boys are more likely to be punished for aggressive behavior by both teachers and parents (31, 32).

Cross-cultural comparisons can be valuable in establishing the extent to which a given pattern of behavior, thought, or emotion is a human universal (2, 19, 33, 34). Norenzayan and Heine (33) have considered criteria for a conclusion of universality, and noted that psychological inclinations can range from clear “nonuniversals” (such as computational logic linked to the use of an abacus) through “accessibility” universals which are functionally the same across cultures (such as hormonal changes and behaviors associated with nursing). The reversal of sex differences in aggression discussed here is quite robust but would likely meet the criteria for what Norenzayan and Heine call “functional universals.” Norenzayan and Heine define a functional universal as reflecting strong underlying psychological mechanisms, but allowing some variation in accessibility across contexts (giving the similarity-attraction principle as one example). In examining the 4 measured categories across 24 countries for sibling versus nonsibling in aggression reported here, the reversed pattern is found in most comparisons, but there are variations in the strength of that reversal. Of course, some variations were found even in reported direct aggression outside the family, for which, in 3 of 96 comparisons for hitting or slapping another child in Sweden and Romania, and for yelling at someone as an adult in Sweden, females reported significantly more direct aggression outside the family than did males.

The study of kin relations has been called a “conceptual hole” in psychological studies of close relationships (which historically focused on relationships between romantic partners or friendships outside the family) (35). The current findings add to a growing literature aiming to fill that hole (15, 16, 36–38). One surprising finding in this area is the relatively high level of aggression between siblings (16, 39, 40). Several studies found that parents reported that between 74 and 90% of their children had engaged in some form of violence with their siblings. And people's own self-reports of aggression toward and from their siblings bear these parents' reports out, with aggression between siblings being higher than that reported toward friends and acquaintances (16). These studies, as well as the present work, are not consistent with evolutionary models based on sexual selection and differential parental investment (which address higher levels of male aggression outside the family), but they are broadly consistent with Trivers (41) theory of sibling rivalry. Although siblings typically share a high proportion of genes, enough to provide constraints on the severity of aggressive acts, each individual shares 100% of his or her genes with himself but only 50% with his or her siblings, thus inclining individuals to compete for a relatively higher portion of parental resources with their sibling(s).

The present work is not without limitations. Behavior in this study was assessed using self-report rather than direct observation or reports from other observers. However, supporting the interpretation that these self-reports may accurately reflect behavior, we replicate classic findings of greater male propensity to direct aggression outside of the family—a finding previously demonstrated not only with self-report but observational and archival data. However, the use of self-report in this context has other potential limitations. One possibility is that males may engage in higher levels of direct aggression toward sisters but be unwilling to admit this due to social desirability concerns. That said, prior work with American samples did not find discrepancies consistent with that interpretation; results were identical whether participants were asked about aggression others had directed toward them or that they had directed toward other (15, 16). Another possibility is that men and women may have different standards for what types of behavior rise to the level “hitting,” for example. It is possible that men and women engaging in the same behavior may differ in whether they interpret it as hostile or playful. However, this interpretation does not fit well with the fact that sex differences in direct aggression toward nonkin were found in nearly every society in this study and in the opposite direction of the few significant sex differences in aggression toward siblings that were observed. Finally, we note that we asked about the frequency of aggressive behaviors in relatively general terms, rather than asking for specific counts or numerical estimates. When surveying individuals about the frequency of behavior, there are tradeoffs associated with each approach. People's accuracy at recalling exact counts of behavior over long spans of time tends to be poor (31). However, people may also differ in how they interpret phrases such as “often” or “many times.” Thus, in the future, it would be useful to conduct observational research or to gather reports from caregivers as this might help allay some of these concerns regarding the interpretation of these self-reported data.

Although the present work cannot definitively answer questions regarding mechanisms underpinning the effects observed, our results are consistent with explanations based on intrafamilial conflict and inclusive fitness and inconsistent with previous accounts rooted in social role theory or sexual selection and differential parental investment (which had been advanced to explain the abundant findings of higher male aggression outside the family). That said, future work should seek to directly test such mechanisms to the extent that it is possible. Future studies could be conducted longitudinally in order to evaluate the veracity of the inclusive fitness theory versus the social role theory more carefully, and others could explore if differences in aggressive behaviors exist between male and female siblings when the target varies in terms of genetic relatedness (e.g. twins, full siblings, half siblings, and biologically unrelated siblings).

## Supplementary Material

pgaf239_Supplementary_Data

## Data Availability

All data and R code are freely available on the Open Science Framework (OSF) at https://osf.io/7239g/ (42)

## Notes

a Target categories were manipulated within-subjects and were collapsed to compare siblings (of both sexes) against both types of nonsiblings (i.e. friends and acquaintances of both sexes). Models that compared all targets and sex of target are included in the online supplement.

b We first considered the full, more complex model that included type of aggression and child versus adult as within-subjects factors in addition to target and sex of participant (details provided in online supplement). After obtaining a significant four-way interaction, we broke the analysis down further as described here to focus on sex differences in each form of aggression against siblings versus nonsiblings.
