# Correction to: Commonly observed sex differences in direct aggression are absent or reversed in sibling contexts

**DOI:** 10.1093/pnasnexus/pgaf293

**Published:** 2025-09-30

This is a **correction** to:

Michael E W Varnum, Amanda P Kirsch, Daniel J Beal, Cari M Pick, Laith Al-Shawaf, Chiara Ambrosio, Maria Teresa Barbato, Oumar Barry, Watcharaporn Boonyasiriwat, Eduard Brandstätter, Suzan Ceylan-Batur, Marco Antonio Correa Varella, Julio Eduardo Cruz, Oana David, Laina Ngom Dieng, Dimitri Dubois, Ana María Fernandez, Silvia Galdi, Oscar Javier Galindo Caballero, Sylvie Graf, Igor Grossmann, David Guzman, Peter Halama, Takeshi Hamamura, Martina Hřebíčková, Ioana Iuga, Lady Javela, Jaewuk Jung, Johannes A Karl, Jinseok P Kim, Michal Kohút, Anthonieta Looman Mafra, Dieynaba Gabrielle Ndiaye, Jiaqing O, Beatriz Perez Sánchez, Eric Roth Unzueta, Muhammad Rizwan, A Timur Sevincer, Eric Skoog, Eunkook M Suh, Daniel Sznycer, Evelina Thunell, Arnaud Tognetti, Ayse K Uskul, Jaroslava Varella Valentova, Yunsuh Nike Wee, Anja Lundkvist Winter, Torin Peter Young, Danilo Zambrano, Anna Ziska, Douglas T Kenrick, Commonly observed sex differences in direct aggression are absent or reversed in sibling contexts, *PNAS Nexus*, Volume 4, Issue 8, August 2025, pgaf239, https://doi.org/10.1093/pnasnexus/pgaf239

This paper has been updated to move author, Jiaqing O's, 2^nd^ affiliation (University of Macau) to the Author Notes section. This is to indicate that this is the author's current affiliation. The first affiliation (Aberystwyth University) is retained in the author byline to indicate that the work for the paper was completed at this institution. The publisher apologizes for the original error.

