# Supplementary material for: Commonly observed sex differences in direct aggression are absent or reversed in sibling contexts
Source: PNAS Nexus. 2025 Aug 26;4(8):pgaf239. doi: 10.1093/pnasnexus/pgaf239 (PMC12378733; doi:10.1093/pnasnexus/pgaf239)
Supplement: pgaf239_Supplementary_Data [file pgaf239_supplementary_data.pdf]

Supplementary Material

Commonly observed Sex Differences in Direct Aggression are Absent or Reversed in Sibling Contexts

Michael E. W. Varnum [1]*, Amanda P. Kirsch [1], Daniel J. Beal [2], Cari M. Pick [3], Laith Al-Shawaf [4], Chiara Ambrosio [5], Maria Teresa Barbato [6], Oumar Barry [7], Watcharaporn Boonyasiriwat [8], Eduard Brandstätter [9], Suzan Ceylan-Batur [10], Marco Antonio Correa Varella [11], Julio Eduardo Cruz [12], Oana David [13], Laina Ngom Dieng [7], Dimitri Dubois [14], Ana María Fernandez [6], Silvia Galdi [5], Oscar Javier Caballero [15], Sylvie Graf [16], Igor Grossmann [17], David Guzman [18], Peter Halama [19], Takeshi Hamamura [20], Martina Hřebíčková [16], Ioana Iuga [13], Lady Javela [21], Jaewuk Jung [22], Johannes A. Karl [23, 24], Jinseok P. Kim [25], Michal Kohút [26], Anthonieta Looman Mafra [11], Dieynaba Gabrielle Ndiaye [7], Jiaqing O [27, 28], Beatriz Perez Sánchez [29, 30], Eric Roth Unzueta [31], Muhammad Rizwan [32], A. Timur Sevincer [33], Eric Skoog [34], Eunkook M. Suh [25], Daniel Sznycer [35], Evelina Thunell [36], Arnaud Tognetti [14, 36], Ayse K. Uskul [37], Jaroslava Varella Valentova [11], Yunsuh N. Wee [35], Anja Lundkvist Winter [36], Torin Peter Young [17], Danilo Zambrano [18], Anna Ziska [38], Douglas T. Kenrick [1]

Appendix A: Sample characteristics and data collection details broken down by country

Table S1. Sample characteristics broken down by country

Country	Usable Sample size for each country	Median number of siblings	Sex	Mean Age (SD)	Ethnicity	Mean Social Status (SD)	Number removed due to failed attention checks	Number removed due to gender other than male/ female
Australia	124	1	22% M<br>78% F	24.0 (8.2)	81% Australian<br>4% New Zealander<br>4% Middle East<br>2% Sub-saharan African<br>5% East Asia<br>10% South Asia<br>1% Central Asia<br>1% West Indies/Carribean<br>1% Central American<br>2% South American	6.4 (1.4)	4	1
Austria	101	N/A	42.3% M<br>57.7% F	28.0 (9.5)	97.9% white<br>2.1% other	5.8 (1.7)	5	3
Bolivia	103	2	34.4% M<br>65.6% F	21.6 (3.5)	21.3% white<br>15.7% mixed<br>1% middle eastern	6.7 (1.4)	20	1

					1% asian<br>28% Aymara or Quechua<br>5% black<br>28% other			
Brazil	259	2	21.6% M<br>78.4% F	41.1 (14.1)	64.2% white<br>11.7% mixed<br>1% indigenous<br>3.7% black<br>3.1% asian<br>0.9% other<br>15.4% pardo	5.8 (1.9)	10	2
Canada	92	1	24.2% M<br>75.8% F	19.7 (3.3)	3.3% black<br>13.2% east Asian<br>6.6% mixed<br>2.2% hispanic<br>3.3% middle eastern<br>22% south Asian<br>10% southeast Asian<br>39.4% white	6.2 (1.5)	13	1
Chile	151	2	27.3% M<br>72.7% F	29.3 (10.2)	39.4% white<br>7.3% mixed<br>11.1% indigenous<br>42.2% other	6.1 (SD = 1.5)	6	1

Colombia	129	1	25.9% M<br>74.1% F	20.7 (3.4)	2% Black<br>6% mixed<br>37.6% white<br>2% indigenous<br>37.6% mestizo<br>14.8% other	5.7 (SD = 1.5)	14	2
Czechia	165	1	14.9% M<br>85.1% F	36.3 (12.6)	85.4% Czech<br>11.2% Slovakian<br>1.7% Ukrainian<br>1.7% Other	6.8 (1.3)	3	0
France	111	2	33.3% M<br>66.7% F	25.9 (10.0)	N/A	6.2 (1.7)	N/A	2
Germany	195	N/A	25.7% M<br>74.3% F	23.4 (6.9)	N/A	7.0 (1.3)	26	1
Italy	170	N/A	38.5% M<br>61.5% F	21.0 (1.9)	100% Italian	5.7 (1.4)	12	0
Korea	126	N/A	32.3% M<br>67.7% F	30.9 (7.6)	100% Korean	6.3(1.4)	22	2
Lebanon	85	2	26.9% M<br>73.1% F	31.2 (8.6)	N/A	6.3 (1.5)	2	0
New Zealand	208	2	18.7% M	19.1 (2.7)	2.6% African<br>11.7% mixed	6.5 (1.6)	4	1

			81.3% F		7.7% east or south asian<br>69.4% european<br>4.6% maori<br>1.4% Pacific Islander<br>2.6% prefer not to say or other			
Pakistan	283	4	24.2% M<br>75.8% F	23.8 (6.8)	1.6% Balouchi<br>0.4% Balti<br>2.9% Karhmiri<br>15.2% other<br>23.8% Pathan<br>3.7% mixed<br>21.7% Punjabi<br>0.4% Sindhi<br>30.3% Urdu	6.7 (2.0)	74	2
Romania	209	1	11.6% M<br>88.4% F	31.5 (11.6)	94.8% Romanian<br>1% Hungarian<br>4.2% mixed	6.7 (1.4)	11	1
Senegal	400	N/A	48.8% M<br>51.2% F	22.2 (2.9)	1.2% Bambara<br>6.8% Joola<br>5.6% Mandingue<br>1.6% Manjack<br>9.6% Other<br>25.2% Peul<br>4% mixed<br>16.8% Sereer	5.9 (2.4)	126	3

					29.2% Wolof			
Slovakia	149	N/A	34.9% M<br>65.1% F	26.2 (8.2)	N/A	6.1 (1.2)	0	0
Spain	106	N/A	30.2 M<br>69.8% F	31.3 (14.2)	N/A	4.2 (1.3)	6	0
Sweden	243	1	16.7% M<br>83.3% F	30.27 (14.15)	N/A	6.2 (2.1)	N/A	3
Thailand	183	N/A	27% M<br>73% F	24 (7.4)	91.2% East/Southeast Asian<br>2.9% mixed<br>2.2% other<br>2.9% south Asian<br>0.8% white	6.5 (1.3)	17	5
Turkey	101	N/A	25.7% M<br>74.3% F	27 (8.5)	94.1% Turkish<br>3.9% Kurdish<br>2% Other	7.0 (1.5)	N/A	1
UK	317	1	45.1% M<br>54.9% F	25.6 (9.6)	76.4% White<br>3.4% mixed<br>5.6% Black<br>2.1% East Asian<br>0.4% Gypsy/Irish Traveler	5.5 (1.7)	7	5

					2.6% Middle eastern/North african<br>2.6% other<br>6.9% South Asian			
USA	126	2	24.3% M<br>75.7% F	20.9 (3.7)	11.2% Asian<br>5.2% Black<br>11.2% More than 0.9% race<br>2.6% Native American<br>13.8% other<br>55.1% white	6.1 (1.4)	4	1

Table S2. **Data collection details by country**

Country/ Society	*Sample Type*	*Collection Dates*	*Survey Language*	*Region of country, if available*	*Translation Procedure*
Australia	University	March-April 2022	English	Western Australia	-
Austria	Online	Feb.-March 2022	German		Translated by 2 translators
Bolivia	Information not available	Information not available	Spanish		Information not available
Brazil	Online	May 2022-April 2023	Portuguese		Translated by 2 translators; back-translated by 2 additional translators; harmonized by 5th translator
Canada	University	2022	English	Ontario	-
	Online	Sep.-Oct. 2022			
Chile	University, Community	July 2020	Spanish	Santiago	Translated; checked by 2nd translator
Colombia	University	2nd semester of 2022	Spanish	Bogotá D. C.	Translated; back-translated by 2nd translator
Czech Republic	University, Community	March-June 2022	Czech		Translated; checked by 2 additional translators
France	University	Dec. 2022-Jan. 2023	French	Montpellier	Translated; back-translated by 2nd translator; checked by 3rd translator
Germany	University	June 2022	German	Hamburg	Translated; checked by second translator
Italy	University	March 2022	Italian	Campania	Translated and back-translated
Lebanon	Online	Feb.-Nov. 2022	English		-
New Zealand	University	March-May 2022	English		-
Pakistan	University, Community	Information not available	English	Islamabad, Karachi, Lahore	-
Romania	University, Community, Online	March-June 2022	Romanian	Cluj	Translated; checked by 2nd translator
Senegal	University	March-April 2022	French		Translated; back-translated by 2nd translator
Slovakia	University, Community	Feb.-March 2022	Slovak	Western Slovakia	Translated by 2 independent translators and then harmonized
South Korea	University	Feb.-May 2022	Korean	Seoul	Translated; checked by 2nd translator
	University	Feb.-March 2022		Seoul	Information not available
Spain	Community	May 2022	Spanish	Asturias	Translated; check by 2 additional translators
Sweden	Community	Oct.-Nov. 2022	Swedish	Stockholm	Translated and back-translated
	University	Dec. 2022-March 2023		Uppsala	Translated
Thailand	University	March 2023	Thai		Translated; back-translated by 2nd translator
Turkey	University, Community	Dec. 2022	Turkish	Ankara	Translated by native speaker; back-translated by 2 other native speakers

United Kingdom	University	Information not available	English	Southeast England	-
	Online	Feb-June 2022			

Note: For each data collection, the Sample Type indicates whether participants were from a university sample, community sample, and/or online paid workforce (e.g., Prolific, Amazon's Mechanical Turk). The city/cities or Region of the country where data collection took place, if applicable, is described. In several countries, multiple data collections occurred and are noted. Approximate start and end dates of data collection for each sample are indicated. The Survey Language indicates the language in which survey materials were administered, and the Translation Procedure describes the process of translation and back-translation and/or translation checking for all non-English materials.

Appendix B: Descriptive statistics and correlations between study variable:

***Table S3*. Descriptive statistics for varying forms of aggression toward siblings and non-siblings**

Aggression Variable	M	SD	Skew	Kurtosis	N (individuals)	N (countries)	ICC(1) Individual	ICC(1) Country
				Aggression Toward Siblings				
Hit	2.00	.880	.58	-.60	4,131	24	.313	.031
Yell	2.79	.909	-.35	-.82	4,124	24	.365	.029
Gossip within Family	1.54	.784	1.35	.82	4,106	24	.436	.065
Gossip outside Family	1.44	.729	1.65	1.89	4,106	24	.451	.061
Report within Family	2.36	.943	.10	-1.04	4,115	24	.386	.031
Report outside Family	1.28	.579	2.34	5.24	4,116	24	.336	.078
				Aggression Toward Non-Siblings				
Hit	1.39	.487	1.52	2.43	4,136	24	.272	.063
Yell	1.91	.718	.57	-.50	4,135	24	.390	.043
Gossip within Family	1.54	.667	1.17	.44	4,097	24	.482	.072
Gossip outside Family	1.61	.702	.97	-.07	4,095	24	.474	.078
Report within Family	1.72	.708	.81	-.24	4,116	24	.464	.043
Report outside Family	1.39	.540	1.57	1.98	4,116	24	.420	.053

Table S4. **Country and individual level correlations between study variables**

		2	3	4	5	6	7	8	9	10	11	12	13
1	Gross Domestic Product	.621	-.732	.552	.468	.018	.151	.405	-.022	-.112	-.187	-.107	-.306
2	Gender Egalitarianism		-.513	.426	.338	-.011	.278	.232	-.052	-.047	-.035	-.299	-.318
3	Cultural Distance			-.649	-.605	.128	.064	-.255	-.035	.097	.087	.193	.350
4	Gossiping outside Family				.984	.509	.610	.619	.539	-.378	-.319	.370	-.803
5	Gossiping within Family			.847		.552	.618	.676	.535	-.399	-.387	.469	-.756
6	Hitting/Slapping			.265	.265		.821	.549	.585	-.064	-.369	.494	-.617
7	Reporting outside Family			.390	.396	.313		.658	.503	-.271	-.208	.491	-.739
8	Reporting within Family			.406	.494	.297	.529		.398	-.450	-.448	.641	-.418
9	Yelling			.342	.354	.572	.336	.411		-.248	-.125	.388	-.630
10	Sex			-.019	-.053	.110	.010	-.158	.033		-.218	-.508	.269
11	Age			-.068	-.063	-.120	-.095	-.150	-.044	.092		-.471	.074
12	Status			-.019	-.024	-.074	-.019	.003	-.050	.013	.068		-.361
13	Number of Siblings			-.044	-.007	-.001	-.020	-.033	.019	.023	.124	-.023	

Note: Correlations above the diagonal are aggregated to the country level (N = 24), with coefficients above .405 significant at $p < .05$. Correlations below the diagonal are within-country (aggregated across conditions to the individual level; Ns range from 4,095–4,136), with coefficients above .031 significant at $p < .05$.

Appendix C: Results from the hypothesized four-factor mixed-effects multilevel regression model

As discussed in the main text, we examined our hypotheses with a mixed-effects multilevel regression model that included within-subject factors of family status (family or non-family member), participant age (child or adult), and type of aggression (hit, yell, gossip within the family, gossip outside of family, report within family, or report outside of family), and the between-subjects factor of participant sex. These factors were nested within the 24 countries examined, creating a three-level random coefficient regression model. Although simpler models presented in the main text were estimated using maximum likelihood, the full four-factor mixed effects multilevel regression model could not be estimated using this approach (i.e., as there were more country-level parameters to be estimated than there were countries). We therefore relied on a Bayesian estimation method for this model and the summary of the model parameters included here is based on this model. More specifically, we used the Bayesian estimation methods included in Mplus v. 8.10, relying on its default priors, using 2 chains in the Markov Chain Monte Carlo estimation with no thinning and specifying a Potential Scale Reduction (PSR) threshold value of 1.05 or less. We first ran the model without constraining the number of iterations and then repeated the estimation after doubling the number of iterations while ensuring that the model still met the PSR threshold value.

One consequence of relying on Bayesian estimation is that the credibility intervals for random effect variances will always not include zero (i.e., because the parameter space of variances cannot be negative), but the interpretation that these intervals are therefore meaningful is not accurate. Instead, it is recommended that the magnitude of the variance be the focus of interpretation, with one suggestion indicating that when the variance component estimate is more than three times its standard error, then it is a notably large effect and likely worth inclusion and further inspection (Asparouhov & Muthen, 2022). We note here that none of the random effect variances reported below met this threshold—a finding that is consistent with the examination of random effect variances reported in the main text. Below, we have included the Mplus input code followed by the output table of parameter estimates.

MPLUS Input File Code

```
TITLE: 4-factor mixed-effects model
DATA:
  FILE IS LongformData.csv;
VARIABLE:

  NAMES ARE IID PS CID AggAct Targ WvO AvC Score
  GDP GEgal CDist;
  USEVARIABLES ARE Score PS TF CA AT1-AT5 TFCA TFAT1 TFAT2 TFAT3
  TFAT4 TFAT5 CAAT1 CAAT2 CAAT3 CAAT4 CAAT5
  TFCAAT1 TFCAAT2 TFCAAT3 TFCAAT4 TFCAAT5;

  MISSING = ALL(-99);
  CLUSTER = CID IID;
  WITHIN ARE TF CA AT1-AT5 TFCA TFAT1 TFAT2 TFAT3
  TFAT4 TFAT5 CAAT1 CAAT2 CAAT3 CAAT4 CAAT5
  TFCAAT1 TFCAAT2 TFCAAT3 TFCAAT4 TFCAAT5;
  BETWEEN ARE (IID) PS ;

ANALYSIS:
TYPE=THREELEVEL RANDOM;
ESTIMATOR = BAYES;
```

```
BITERATIONS = 100000(12000); ! First run finished in 6000 iterations
PROCESSORS = 8;

MODEL:

! A = Target in Family or Not in Family (sib/non-sib)
! B = Participant Age (child/adult)
! C1 = Dummy for Type of Aggression (Hit/Yell)
! C2 = Dummy for Type of Agg (Hit/Goss within fam)
! C3 = Dummy for Type of Agg (Hit/Goss outside fam)
! C4 = Dummy for Type of Agg (Hit/Rep within fam)
! C5 = Dummy for Type of Agg (Hit/Rep outside fam)
! D = Participant Sex (female/male)

%within%

! Main Effects (within-subjects)
A  | Score ON TF; ! Main Effect of Targ Fam/NFam
B  | Score ON CA; ! Main Effect of Part Age
C1 | Score ON AT1; ! Main Effect of Type of Agg (Hit v Yell)
C2 | Score ON AT2; ! Main Effect of Type of Agg (Hit v Goss within fam)
C3 | Score ON AT3; ! Main Effect of Type of Agg (Hit v Goss outside fam)
C4 | Score ON AT4; ! Main Effect of Type of Agg (Hit v Rep within fam)
C5 | Score ON AT5; ! Main Effect of Type of Agg (Hit v Rep outside fam)

! Two-Way Interactions (within-subjects)
AB  | Score ON TFCA  ;
! Targ Fam/NFam x Part Age
AC1 | Score ON TFAT1 ;
! Targ Fam/NFam x Type of Agg (Hit v Yell)
AC2 | Score ON TFAT2 ;
! Targ Fam/NFam x Type of Agg (Hit v Goss within fam)
AC3 | Score ON TFAT3 ;
! Targ Fam/NFam x Type of Agg (Hit v Goss outside fam)
AC4 | Score ON TFAT4 ;
! Targ Fam/NFam x Type of Agg (Hit v Rep within fam)
AC5 | Score ON TFAT5 ;
! Targ Fam/NFam x Type of Agg (Hit v Rep outside fam)
BC1 | Score ON CAAT1 ;
! Part Age x Type of Agg (Hit v Yell)
BC2 | Score ON CAAT2 ;
! Part Age x Type of Agg (Hit v Goss within fam)
BC3 | Score ON CAAT3 ;
! Part Age x Type of Agg (Hit v Goss outside fam)
BC4 | Score ON CAAT4 ;
! Part Age x Type of Agg (Hit v Rep within fam)
BC5 | Score ON CAAT5 ;
! Part Age x Type of Agg (Hit v Rep outside fam)

! Three-Way Interactions (within-subjects)

ABC1 | Score ON TFCAAT1;
! Targ Fam/NFam x Part Age x Type Agg (Hit v Yell)
ABC2 | Score ON TFCAAT2;
! Targ Fam/NFam x Part Age x Type Agg (Hit v Goss within fam)
ABC3 | Score ON TFCAAT3;
! Targ Fam/NFam x Part Age x Type Agg (Hit v Goss outside fam)
ABC4 | Score ON TFCAAT4;
! Targ Fam/NFam x Part Age x Type Agg (Hit v Rep within fam)
ABC5 | Score ON TFCAAT5;
! Targ Fam/NFam x Part Age x Type Agg (Hit v Rep outside fam)


%between IID%

D | Score ON PS; ! Main Effect of Part Sex

! Two-Way Interactions (between x within)

AD  | A  ON PS;
! Targ Fam/NFam x Part Sex
```

```
BD  | B  ON PS;
! Part Age x Part Sex
DD1 | C1 ON PS;
! Type of Agg (Hit v Yell) x Part Sex
DD2 | C2 ON PS;
! Type of Agg (Hit v Goss within fam) x Part Sex
DD3 | C3 ON PS;
! Type of Agg (Hit v Goss outside fam) x Part Sex
DD4 | C4 ON PS;
! Type of Agg (Hit v Rep within fam) x Part Sex
DD5 | C5 ON PS;
! Type of Agg (Hit v Rep outside fam) x Part Sex

! Three-way Interactions (between x within x within)

ABD  | AB  ON PS;
! Targ Fam/NFam x Part Age x Part Sex
AC1D | AC1 ON PS;
! Targ Fam/NFam x Type of Agg (Hit v Yell) x Part Sex
AC2D | AC2 ON PS;
! Targ Fam/NFam x Type of Agg (Hit v Goss within fam)  x Part Sex
AC3D | AC3 ON PS;
! Targ Fam/NFam x Type of Agg (Hit v Goss outside fam) x Part Sex
AC4D | AC4 ON PS;
! Targ Fam/NFam x Type of Agg (Hit v Rep within fam) x Part Sex
AC5D | AC5 ON PS;
! Targ Fam/NFam x Type of Agg (Hit v Rep outside fam) x Part Sex
BC1D | BC1 ON PS;
! Part Age x Type of Agg (Hit v Yell) x Part Sex
BC2D | BC2 ON PS;
! Part Age x Type of Agg (Hit v Goss within fam)  x Part Sex
BC3D | BC3 ON PS;
! Part Age x Type of Agg (Hit v Goss outside fam) x Part Sex
BC4D | BC4 ON PS;
! Part Age x Type of Agg (Hit v Rep within fam) x Part Sex
BC5D | BC5 ON PS;
! Part Age x Type of Agg (Hit v Rep outside fam) x Part Sex

! Four-Way Interactions (within x within x within x between)

ABC1D | ABC1 ON PS;
! Targ Fam/NFam x Part Age x Type Agg (Hit v Yell) x Part Sex
ABC2D | ABC2 ON PS;
! Targ Fam/NFam x Part Age x Type Agg (Hit v Goss within fam) x Part Sex
ABC3D | ABC3 ON PS;
! Targ Fam/NFam x Part Age x Type Agg (Hit v Goss outside fam) x Part Sex
ABC4D | ABC4 ON PS;
! Targ Fam/NFam x Part Age x Type Agg (Hit v Rep within fam) x Part Sex
ABC5D | ABC5 ON PS;
! Targ Fam/NFam x Part Age x Type Agg (Hit v Rep outside fam) x Part Sex


%between CID%

D-ABC5D;

OUTPUT: TECH8;

DEFINE:

GDP = GDP/1000; ! Transformed to avoid "variance too large" errors

! Recoded Main Effect Variables

! Needed to determine whether reporting and gossiping is within v outside fam
IF (WvO EQ 1) THEN WvO = 1; ! Within is 0, Outside is 1
IF (WvO EQ 2) THEN WvO = 0; ! Within is 0, Outside is 1

IF (Targ EQ 1) THEN TF = 0; ! Fam is 0, NFam is 1
IF (Targ EQ 2) THEN TF = 1; ! Fam is 0, NFam is 1
IF (Targ EQ 3) THEN TF = 1; ! Fam is 0, NFam is 1
```

```
IF (Targ EQ 4) THEN TF = 1; ! Fam is 0, NFam is 1
IF (Targ EQ 5) THEN TF = 1; ! Fam is 0, NFam is 1
IF (Targ EQ 6) THEN TF = 0; ! Fam is 0, NFam is 1

IF (AvC EQ 1) THEN CA = 1; ! Recode so Child is 0, Adult is 1
IF (AvC EQ 2) THEN CA = 0; ! Recode so Child is 0, Adult is 1

IF (AggAct EQ 2) THEN AT1 = 0; ! Hitting is referent
IF (AggAct EQ 2) THEN AT2 = 0; ! Hitting is referent
IF (AggAct EQ 2) THEN AT3 = 0; ! Hitting is referent
IF (AggAct EQ 2) THEN AT4 = 0; ! Hitting is referent
IF (AggAct EQ 2) THEN AT5 = 0; ! Hitting is referent

IF (AggAct EQ 4) THEN AT1 = 1; ! Yelling
IF (AggAct EQ 4) THEN AT2 = 0; ! Yelling
IF (AggAct EQ 4) THEN AT3 = 0; ! Yelling
IF (AggAct EQ 4) THEN AT4 = 0; ! Yelling
IF (AggAct EQ 4) THEN AT5 = 0; ! Yelling

IF (AggAct EQ 1 AND WvO EQ 0) THEN AT1 = 0; ! Gossiping within fam
IF (AggAct EQ 1 AND WvO EQ 0) THEN AT2 = 1; ! Gossiping within fam
IF (AggAct EQ 1 AND WvO EQ 0) THEN AT3 = 0; ! Gossiping within fam
IF (AggAct EQ 1 AND WvO EQ 0) THEN AT4 = 0; ! Gossiping within fam
IF (AggAct EQ 1 AND WvO EQ 0) THEN AT5 = 0; ! Gossiping within fam

IF (AggAct EQ 1 AND WvO EQ 1) THEN AT1 = 0; ! Gossiping outside fam
IF (AggAct EQ 1 AND WvO EQ 1) THEN AT2 = 0; ! Gossiping outside fam
IF (AggAct EQ 1 AND WvO EQ 1) THEN AT3 = 1; ! Gossiping outside fam
IF (AggAct EQ 1 AND WvO EQ 1) THEN AT4 = 0; ! Gossiping outside fam
IF (AggAct EQ 1 AND WvO EQ 1) THEN AT5 = 0; ! Gossiping outside fam

IF (AggAct EQ 3 AND WvO EQ 0) THEN AT1 = 0; ! Reporting within fam
IF (AggAct EQ 3 AND WvO EQ 0) THEN AT2 = 0; ! Reporting within fam
IF (AggAct EQ 3 AND WvO EQ 0) THEN AT3 = 0; ! Reporting within fam
IF (AggAct EQ 3 AND WvO EQ 0) THEN AT4 = 1; ! Reporting within fam
IF (AggAct EQ 3 AND WvO EQ 0) THEN AT5 = 0; ! Reporting within fam

IF (AggAct EQ 3 AND WvO EQ 1) THEN AT1 = 0; ! Reporting outside fam
IF (AggAct EQ 3 AND WvO EQ 1) THEN AT2 = 0; ! Reporting outside fam
IF (AggAct EQ 3 AND WvO EQ 1) THEN AT3 = 0; ! Reporting outside fam
IF (AggAct EQ 3 AND WvO EQ 1) THEN AT4 = 0; ! Reporting outside fam
IF (AggAct EQ 3 AND WvO EQ 1) THEN AT5 = 1; ! Reporting outside fam


! Calculated Two-Way Interaction Variables
TFCA  = TF*CA;
TFAT1 = TF*AT1;
TFAT2 = TF*AT2;
TFAT3 = TF*AT3;
TFAT4 = TF*AT4;
TFAT5 = TF*AT5;
CAAT1 = CA*AT1;
CAAT2 = CA*AT2;
CAAT3 = CA*AT3;
CAAT4 = CA*AT4;
CAAT5 = CA*AT5;

! Calculated Three-Way Interaction Variables
TFCAAT1 = TF*CA*AT1;
TFCAAT2 = TF*CA*AT2;
TFCAAT3 = TF*CA*AT3;
TFCAAT4 = TF*CA*AT4;
TFCAAT5 = TF*CA*AT5;
```

MPLUS Output Table of Parameter Estimates

MODEL RESULTS

	Estimate	Posterior S.D.	One-Tailed P-Value	95% C.I. Lower 2.5%	95% C.I. Upper 2.5%	Significance
**Within Level**						
Residual Variances						
SCORE	0.368	0.001	0.000	0.366	0.371	*
**Between IID Level**						
Residual Variances						
SCORE	0.184	0.006	0.000	0.174	0.195	*
A	0.097	0.004	0.000	0.091	0.104	*
B	0.064	0.002	0.000	0.060	0.069	*
C1	0.168	0.007	0.000	0.155	0.183	*
C2	0.189	0.007	0.000	0.177	0.203	*
C3	0.171	0.007	0.000	0.159	0.185	*
C4	0.244	0.009	0.000	0.227	0.263	*
C5	0.112	0.005	0.000	0.103	0.122	*
AB	0.014	0.002	0.000	0.010	0.017	*
AC1	0.183	0.008	0.000	0.167	0.199	*
AC2	0.052	0.005	0.000	0.043	0.063	*
AC3	0.102	0.006	0.000	0.090	0.115	*
AC4	0.196	0.009	0.000	0.180	0.215	*
AC5	0.040	0.004	0.000	0.031	0.049	*
BC1	0.062	0.005	0.000	0.052	0.073	*
BC2	0.005	0.003	0.000	0.002	0.013	*
BC3	0.012	0.005	0.000	0.005	0.022	*
BC4	0.069	0.006	0.000	0.058	0.080	*
BC5	0.002	0.001	0.000	0.001	0.005	*
ABC1	0.010	0.005	0.000	0.003	0.022	*
ABC2	0.002	0.002	0.000	0.001	0.007	*
ABC3	0.009	0.006	0.000	0.001	0.020	*
ABC4	0.027	0.008	0.000	0.011	0.042	*
ABC5	0.001	0.001	0.000	0.001	0.006	*
**Between CID Level**						
Means						
SCORE	2.262	0.049	0.000	2.164	2.359	*
A	-0.871	0.040	0.000	-0.950	-0.793	*
B	-0.656	0.042	0.000	-0.741	-0.573	*
C1	0.811	0.052	0.000	0.709	0.913	*
C2	-0.647	0.051	0.000	-0.747	-0.547	*
C3	-0.774	0.045	0.000	-0.865	-0.686	*
C4	0.391	0.049	0.000	0.293	0.488	*
C5	-0.967	0.048	0.000	-1.064	-0.871	*
AB	0.485	0.030	0.000	0.424	0.546	*
AC1	-0.240	0.040	0.000	-0.321	-0.163	*
AC2	0.877	0.047	0.000	0.785	0.969	*
AC3	1.076	0.053	0.000	0.971	1.181	*
AC4	0.116	0.044	0.006	0.029	0.203	*
AC5	1.030	0.043	0.000	0.946	1.117	*
BC1	0.131	0.030	0.000	0.071	0.189	*
BC2	0.494	0.033	0.000	0.430	0.559	*
BC3	0.562	0.038	0.000	0.488	0.639	*
BC4	0.092	0.035	0.007	0.021	0.161	*
BC5	0.588	0.037	0.000	0.515	0.662	*
ABC1	-0.208	0.029	0.000	-0.265	-0.150	*
ABC2	-0.433	0.031	0.000	-0.495	-0.372	*
ABC3	-0.505	0.035	0.000	-0.573	-0.437	*
ABC4	-0.177	0.031	0.000	-0.237	-0.114	*
ABC5	-0.577	0.034	0.000	-0.644	-0.512	*
D	-0.166	0.031	0.000	-0.225	-0.104	*
AD	0.365	0.034	0.000	0.297	0.430	*
BD	0.147	0.031	0.000	0.085	0.206	*

DD1	-0.042	0.035	0.116	-0.112	0.027	
DD2	0.118	0.041	0.003	0.036	0.198	*
DD3	0.116	0.041	0.002	0.038	0.199	*
DD4	-0.090	0.045	0.024	-0.176	-0.001	*
DD5	0.161	0.035	0.000	0.091	0.227	*
ABD	-0.197	0.033	0.000	-0.257	-0.129	*
AC1D	-0.032	0.041	0.211	-0.115	0.047	
AC2D	-0.408	0.041	0.000	-0.487	-0.327	*
AC3D	-0.341	0.044	0.000	-0.426	-0.255	*
AC4D	-0.339	0.048	0.000	-0.433	-0.245	*
AC5D	-0.347	0.038	0.000	-0.419	-0.269	*
BC1D	-0.132	0.041	0.002	-0.213	-0.050	*
BC2D	-0.139	0.040	0.000	-0.215	-0.058	*
BC3D	-0.125	0.039	0.000	-0.201	-0.052	*
BC4D	-0.124	0.043	0.003	-0.206	-0.039	*
BC5D	-0.157	0.041	0.000	-0.233	-0.072	*
ABC1D	0.183	0.046	0.000	0.092	0.273	*
ABC2D	0.219	0.047	0.000	0.123	0.304	*
ABC3D	0.185	0.046	0.000	0.097	0.278	*
ABC4D	0.227	0.047	0.000	0.130	0.316	*
ABC5D	0.207	0.049	0.000	0.108	0.301	*
Variances						
SCORE	0.049	0.019	0.000	0.027	0.099	*
A	0.030	0.012	0.000	0.016	0.062	*
B	0.034	0.014	0.000	0.018	0.071	*
C1	0.052	0.021	0.000	0.028	0.107	*
C2	0.049	0.020	0.000	0.026	0.103	*
C3	0.038	0.016	0.000	0.020	0.081	*
C4	0.047	0.019	0.000	0.024	0.097	*
C5	0.046	0.018	0.000	0.024	0.095	*
AB	0.014	0.006	0.000	0.007	0.031	*
AC1	0.026	0.011	0.000	0.012	0.056	*
AC2	0.041	0.017	0.000	0.022	0.085	*
AC3	0.055	0.022	0.000	0.029	0.114	*
AC4	0.034	0.015	0.000	0.017	0.073	*
AC5	0.035	0.015	0.000	0.018	0.073	*
BC1	0.009	0.006	0.000	0.003	0.024	*
BC2	0.013	0.007	0.000	0.006	0.032	*
BC3	0.022	0.010	0.000	0.010	0.050	*
BC4	0.017	0.008	0.000	0.008	0.039	*
BC5	0.021	0.010	0.000	0.010	0.047	*
ABC1	0.005	0.005	0.000	0.001	0.018	*
ABC2	0.008	0.006	0.000	0.002	0.023	*
ABC3	0.013	0.008	0.000	0.004	0.033	*
ABC4	0.008	0.005	0.000	0.002	0.021	*
ABC5	0.011	0.006	0.000	0.003	0.027	*
D	0.005	0.005	0.000	0.001	0.020	*
AD	0.009	0.006	0.000	0.003	0.025	*
BD	0.002	0.002	0.000	0.001	0.009	*
DD1	0.004	0.004	0.000	0.001	0.016	*
DD2	0.011	0.009	0.000	0.002	0.034	*
DD3	0.012	0.009	0.000	0.003	0.037	*
DD4	0.016	0.011	0.000	0.003	0.046	*
DD5	0.004	0.004	0.000	0.001	0.017	*
ABD	0.002	0.002	0.000	0.001	0.007	*
AC1D	0.006	0.007	0.000	0.001	0.027	*
AC2D	0.010	0.008	0.000	0.002	0.032	*
AC3D	0.014	0.010	0.000	0.003	0.041	*
AC4D	0.016	0.012	0.000	0.004	0.048	*
AC5D	0.005	0.005	0.000	0.001	0.018	*
BC1D	0.003	0.003	0.000	0.001	0.011	*
BC2D	0.002	0.002	0.000	0.001	0.007	*
BC3D	0.002	0.002	0.000	0.001	0.008	*
BC4D	0.003	0.003	0.000	0.001	0.012	*
BC5D	0.002	0.002	0.000	0.001	0.009	*
ABC1D	0.003	0.003	0.000	0.001	0.013	*
ABC2D	0.002	0.003	0.000	0.001	0.010	*
ABC3D	0.003	0.003	0.000	0.001	0.012	*
ABC4D	0.003	0.003	0.000	0.001	0.013	*
ABC5D	0.005	0.005	0.000	0.001	0.018	*

Appendix D: Model Comparisons for Country-Level Variation

***Table S5.* Comparing country-level variation in effects by age and type of sibling aggression**

Type of Aggression	Parameters	DIC	ΔDIC
As Adult, Main Effect of Participant Sex			
Hit/Slap (with country-level variance)	6	13665.49	
Hit/Slap (country-level variance set to 0)	5	13653.32	-12.17
Yell (with country-level variance)	6	14694.21	
Yell (country-level variance set to 0)	5	14683.87	-10.34
Gossip within (with country-level variance)	6	12098.91	
Gossip within (country-level variance set to 0)	5	12085.51	-13.40
Gossip outside (with country-level variance)	6	11766.43	
Gossip outside (country-level variance set to 0)	5	11747.38	-19.05
Report within (with country-level variance)	6	13911.14	
Report within (country-level variance set to 0)	5	13912.08	.94
Report outside (with country-level variance)	6	10298.88	
Report outside (country-level variance set to 0)	5	10286.89	-11.99
As Child, Main Effect of Participant Sex			
Hit/Slap (with country-level variance)	6	15201.60	
Hit/Slap (country-level variance set to 0)	5	15202.92	1.32
Yell (with country-level variance)	6	14340.56	
Yell (country-level variance set to 0)	5	14330.75	-9.81
Gossip within (with country-level variance)	6	12976.10	
Gossip within (country-level variance set to 0)	5	12990.35	14.25
Gossip outside (with country-level variance)	6	12069.34	
Gossip outside (country-level variance set to 0)	5	12081.21	11.87
Report within (with country-level variance)	6	14626.05	
Report within (country-level variance set to 0)	5	14613.13	-12.92
Report outside (with country-level variance)	6	11103.88	
Report outside (country-level variance set to 0)	5	11078.21	-25.67
As Adult, Sibling/Non-Sibling x Sex			
Hit/Slap (with country-level variance)	11	37623.364	
Hit/Slap (country-level variance set to 0)	10	37635.957	12.59
Yell (with country-level variance)	11	45521.064	
Yell (country-level variance set to 0)	10	45527.76	6.70
As Child, Sibling/Non-Sibling x Sex			
Hit/Slap (with country-level variance)	11	45310.451	
Hit/Slap (country-level variance set to 0)	10	45252.751	-57.70
Yell (with country-level variance)	11	47206.675	
Yell (country-level variance set to 0)	10	47203.373	-3.30

Appendix E: Results from full five-factor mixed-effects multilevel regression model

Although we did not have hypotheses involving target sex, our design did allow us the possibility of exploring this factor. To do so, we examined a mixed-effect regression model incorporating all five factors manipulated or measured in our design, nested within each country. Specifically, we regressed the level of aggression on participant sex (female or male; measured between-subjects), participant age (child or adult; manipulated within-subjects), family status of the target (family or non-family member; manipulated within-subjects), sex of the target (female or male; manipulated within-subjects), and type of aggression (hit, yell, gossip within family, gossip outside of family, report within family, or report outside of family; manipulated within-subjects and assessed using dummy codes with hit as the referent category). All of these factors were nested within the 24 countries. Consequently, the model estimated a very large number of random effects and, as with our primary model, precluded relying on traditional maximum likelihood estimation (i.e., convergence issues prevented the simultaneous estimation of all random effects). Furthermore, although we again relied on the Bayesian estimation approach described in Appendix C, the model experienced difficulties in estimating some of the random effect variance parameters. Based on the parameter estimates for the random effect variances involving participant sex from the hypothesized model in Appendix C, we inferred that the estimation difficulties arose due to trying to estimate these very small but non-negative variance parameters. Because the interest in examining the full five-factor model involved an exploration of the fixed effects involving target sex, we decided to fix these random effect variance estimates to a small value (i.e., .01) to assist in estimation of the other model parameters (Asparouhov & Muthen, 2022).

MPLUS Input File Code

```
TITLE: Full 5-factor mixed-effects model
DATA:
  FILE IS LongformData.csv;
VARIABLE:

  NAMES ARE IID PS CID AggAct Targ WvO AvC Score
  GDP GEgal CDist;

  USEVARIABLES ARE Score PS TF TS CA AT1-AT5 TFTS TFCA TFAT1 TFAT2 TFAT3
  TFAT4 TFAT5 TSCA TSAT1 TSAT2 TSAT3 TSAT4 TSAT5 CAAT1 CAAT2 CAAT3 CAAT4 CAAT5
  TFTSCA TFTSAT1 TFTSAT2 TFTSAT3 TFTSAT4 TFTSAT5 TSCAAT1 TSCAAT2 TSCAAT3 TSCAAT4
  TSCAAT5 TFCAAT1 TFCAAT2 TFCAAT3 TFCAAT4 TFCAAT5
  TFTSCAA1 TFTSCAA2 TFTSCAA3 TFTSCAA4 TFTSCAA5;

  MISSING = ALL(-99);
  CLUSTER = CID IID;
  WITHIN ARE TF TS CA AT1-AT5 TFTS TFCA TFAT1 TFAT2 TFAT3
  TFAT4 TFAT5 TSCA TSAT1 TSAT2 TSAT3 TSAT4 TSAT5 CAAT1 CAAT2 CAAT3 CAAT4 CAAT5
  TFTSCA TFTSAT1 TFTSAT2 TFTSAT3 TFTSAT4 TFTSAT5 TSCAAT1 TSCAAT2 TSCAAT3 TSCAAT4
  TSCAAT5 TFCAAT1 TFCAAT2 TFCAAT3 TFCAAT4 TFCAAT5
  TFTSCAA1 TFTSCAA2 TFTSCAA3 TFTSCAA4 TFTSCAA5;
  BETWEEN ARE (IID) PS;

ANALYSIS:
TYPE=THREELEVEL RANDOM;
ESTIMATOR = BAYES;
BITERATIONS = 100000(8800); ! initial run was 4400
PROCESSORS = 8;

MODEL:
! A = Target Family or Not in Family (sib/non-sib)
```

```
! B = Target Sex (female/male)
! C = Participant Age (child/adult)
! D1 = Type of Aggression (Hit/Yell)
! D2 = Type of Agg (Hit/Goss within fam)
! D3 = Type of Agg (Hit/Goss outside fam
! D4 = Type of Agg (Hit/Rep within fam)
! D5 = Type of Agg (Hit/Rep outside fam)
! E = Participant Sex (female/male)

%within%

! Main Effects (within-subjects)
A  | Score ON TF; ! Main Effect of Targ Fam/NFam
B  | Score ON TS; ! Main Effect of Targ Sex
C  | Score ON CA; ! Main Effect of Part Age
D1 | Score ON AT1; ! Main Effect of Type of Agg (Hit v Yell)
D2 | Score ON AT2; ! Main Effect of Type of Agg (Hit v Goss within fam)
D3 | Score ON AT3; ! Main Effect of Type of Agg (Hit v Goss outside fam)
D4 | Score ON AT4; ! Main Effect of Type of Agg (Hit v Rep within fam)
D5 | Score ON AT5; ! Main Effect of Type of Agg (Hit v Rep outside fam)

! Two-Way Interactions (within-subjects)
AB  | Score ON TFTS  ;
! Targ Fam/NFam x Targ Sex
AC  | Score ON TFCA  ;
! Targ Fam/NFam x Part Age
AD1 | Score ON TFAT1 ;
! Targ Fam/NFam x Type of Agg (Hit v Yell)
AD2 | Score ON TFAT2 ;
! Targ Fam/NFam x Type of Agg (Hit v Goss within fam)
AD3 | Score ON TFAT3 ;
! Targ Fam/NFam x Type of Agg (Hit v Goss outside fam)
AD4 | Score ON TFAT4 ;
! Targ Fam/NFam x Type of Agg (Hit v Rep within fam)
AD5 | Score ON TFAT5 ;
! Targ Fam/NFam x Type of Agg (Hit v Rep outside fam)
BC  | Score ON TSCA  ;
! Targ Sex x Part Age
BD1 | Score ON TSAT1 ;
! Targ Sex x Type of Agg (Hit v Yell)
BD2 | Score ON TSAT2 ;
! Targ Sex x Type of Agg (Hit v Goss within fam)
BD3 | Score ON TSAT3 ;
! Targ Sex x Type of Agg (Hit v Goss outside fam)
BD4 | Score ON TSAT4 ;
! Targ Sex x Type of Agg (Hit v Rep within fam)
BD5 | Score ON TSAT5 ;
! Targ Sex x Type of Agg (Hit v Rep outside fam)
CD1 | Score ON CAAT1 ;
! Part Age x Type of Agg (Hit v Yell)
CD2 | Score ON CAAT2 ;
! Part Age x Type of Agg (Hit v Goss within fam)
CD3 | Score ON CAAT3 ;
! Part Age x Type of Agg (Hit v Goss outside fam)
CD4 | Score ON CAAT4 ;
! Part Age x Type of Agg (Hit v Rep within fam)
CD5 | Score ON CAAT5 ;
! Part Age x Type of Agg (Hit v Rep outside fam)

! Three-Way Interactions (within-subjects)

ABC  | Score ON TFTSCA ;
! Targ Fam/NFam x Targ Sex x Part Age
ABD1 | Score ON TFTSAT1;
! Targ Fam/NFam x Targ Sex x Type Agg (Hit v Yell)
ABD2 | Score ON TFTSAT2;
! Targ Fam/NFam x Targ Sex x Type Agg (Hit v Goss within fam)
ABD3 | Score ON TFTSAT3;
! Targ Fam/NFam x Targ Sex x Type Agg (Hit v Goss outside fam)
ABD4 | Score ON TFTSAT4;
! Targ Fam/NFam x Targ Sex x Type Agg (Hit v Rep within fam)
```

```
ABD5 | Score ON TFTSAT5;
! Targ Fam/NFam x Targ Sex x Type Agg (Hit v Rep outside fam)
ACD1 | Score ON TFCAAT1;
! Targ Fam/NFam x Part Age x Type Agg (Hit v Yell)
ACD2 | Score ON TFCAAT2;
! Targ Fam/NFam x Part Age x Type Agg (Hit v Goss within fam)
ACD3 | Score ON TFCAAT3;
! Targ Fam/NFam x Part Age x Type Agg (Hit v Goss outside fam)
ACD4 | Score ON TFCAAT4;
! Targ Fam/NFam x Part Age x Type Agg (Hit v Rep within fam)
ACD5 | Score ON TFCAAT5;
! Targ Fam/NFam x Part Age x Type Agg (Hit v Rep outside fam)
BCD1 | Score ON TSCAAT1;
! Targ Sex x Part Age x Type Agg (Hit v Yell)
BCD2 | Score ON TSCAAT2;
! Targ Sex x Part Age x Type Agg (Hit v Goss within fam)
BCD3 | Score ON TSCAAT3;
! Targ Sex x Part Age x Type Agg (Hit v Goss outside fam)
BCD4 | Score ON TSCAAT4;
! Targ Sex x Part Age x Type Agg (Hit v Rep within fam)
BCD5 | Score ON TSCAAT5;
! Targ Sex x Part Age x Type Agg (Hit v Rep outside fam)


! Four-Way Interactions (within-subjects)

ABCD1 | Score ON TFTSCAA1;
! Targ Fam/NFam x Targ Sex x Part Age x Type Agg (Hit v Yell)
ABCD2 | Score ON TFTSCAA2;
! Targ Fam/NFam x Targ Sex x Part Age x Type Agg (Hit v Goss within fam)
ABCD3 | Score ON TFTSCAA3;
! Targ Fam/NFam x Targ Sex x Part Age x Type Agg (Hit v Goss outside fam)
ABCD4 | Score ON TFTSCAA4;
! Targ Fam/NFam x Targ Sex x Part Age x Type Agg (Hit v Rep within fam)
ABCD5 | Score ON TFTSCAA5;
! Targ Fam/NFam x Targ Sex x Part Age x Type Agg (Hit v Rep outside fam)

%between IID%

E | Score ON PS; ! Main Effect of Part Sex

! Two-Way Interactions (between x within)

AE  | A  ON PS;
! Targ Fam/NFam x Part Sex
BE  | B  ON PS;
! Targ Sex x Part Sex
CE  | C  ON PS;
! Part Age x Part Sex
DE1 | D1 ON PS;
! Type of Agg (Hit v Yell) x Part Sex
DE2 | D2 ON PS;
! Type of Agg (Hit v Goss within fam) x Part Sex
DE3 | D3 ON PS;
! Type of Agg (Hit v Goss outside fam) x Part Sex
DE4 | D4 ON PS;
! Type of Agg (Hit v Rep within fam) x Part Sex
DE5 | D5 ON PS;
! Type of Agg (Hit v Rep outside fam) x Part Sex

! Three-way Interactions (between x within x within)

ABE  | AB  ON PS;
! Targ Fam/NFam x Targ Sex x Part Sex
ACE  | AC  ON PS;
! Targ Fam/NFam x Part Age x Part Sex
AD1E | AD1 ON PS;
! Targ Fam/NFam x Type of Agg (Hit v Yell) x Part Sex
AD2E | AD2 ON PS;
! Targ Fam/NFam x Type of Agg (Hit v Goss within fam)  x Part Sex
AD3E | AD3 ON PS;
```

```
! Targ Fam/NFam x Type of Agg (Hit v Goss outside fam) x Part Sex
AD4E | AD4 ON PS;
! Targ Fam/NFam x Type of Agg (Hit v Rep within fam) x Part Sex
AD5E | AD5 ON PS;
! Targ Fam/NFam x Type of Agg (Hit v Rep outside fam) x Part Sex
BCE  | BC  ON PS;
! Targ Sex x Part Age x Part Sex
BD1E | BD1 ON PS;
! Targ Sex x Type of Agg (Hit v Yell) x Part Sex
BD2E | BD2 ON PS;
! Targ Sex x Type of Agg (Hit v Goss within fam)  x Part Sex
BD3E | BD3 ON PS;
! Targ Sex x Type of Agg (Hit v Goss outside fam) x Part Sex
BD4E | BD4 ON PS;
! Targ Sex x Type of Agg (Hit v Rep within fam) x Part Sex
BD5E | BD5 ON PS;
! Targ Sex x Type of Agg (Hit v Rep outside fam) x Part Sex
CD1E | CD1 ON PS;
! Part Age x Type of Agg (Hit v Yell) x Part Sex
CD2E | CD2 ON PS;
! Part Age x Type of Agg (Hit v Goss within fam)  x Part Sex
CD3E | CD3 ON PS;
! Part Age x Type of Agg (Hit v Goss outside fam) x Part Sex
CD4E | CD4 ON PS;
! Part Age x Type of Agg (Hit v Rep within fam) x Part Sex
CD5E | CD5 ON PS;
! Part Age x Type of Agg (Hit v Rep outside fam) x Part Sex

! Four-Way Interactions (between x within x within x within)

ABCE  | ABC  ON PS;
! Targ Fam/NFam x Targ Sex x Part Age x Part Sex
ABD1E | ABD1 ON PS;
! Targ Fam/NFam x Targ Sex x Type of Agg (Hit v Yell) x Part Sex
ABD2E | ABD2 ON PS;
! Targ Fam/NFam x Targ Sex x Type of Agg (Hit v Goss within fam) x Part Sex
ABD3E | ABD3 ON PS;
! Targ Fam/NFam x Targ Sex x Type of Agg (Hit v Goss outside fam) x Part Sex
ABD4E | ABD4 ON PS;
! Targ Fam/NFam x Targ Sex x Type of Agg (Hit v Rep within fam) x Part Sex
ABD5E | ABD5 ON PS;
! Targ Fam/NFam x Targ Sex x Type of Agg (Hit v Rep outside fam) x Part Sex
ACD1E | ACD1 ON PS;
! Targ Fam/NFam x Part Age x Type Agg (Hit v Yell) x Part Sex
ACD2E | ACD2 ON PS;
! Targ Fam/NFam x Part Age x Type Agg (Hit v Goss within fam) x Part Sex
ACD3E | ACD3 ON PS;
! Targ Fam/NFam x Part Age x Type Agg (Hit v Goss outside fam) x Part Sex
ACD4E | ACD4 ON PS;
! Targ Fam/NFam x Part Age x Type Agg (Hit v Rep within fam) x Part Sex
ACD5E | ACD5 ON PS;
! Targ Fam/NFam x Part Age x Type Agg (Hit v Rep outside fam) x Part Sex
BCD1E | BCD1 ON PS;
! Targ Sex x Part Age x Type of Agg (Hit v Yell) x Part Sex
BCD2E | BCD2 ON PS;
! Targ Sex x Part Age x Type of Agg (Hit v Goss within fam) x Part Sex
BCD3E | BCD3 ON PS;
! Targ Sex x Part Age x Type of Agg (Hit v Goss outside fam) x Part Sex
BCD4E | BCD4 ON PS;
! Targ Sex x Part Age x Type of Agg (Hit v Rep within fam) x Part Sex
BCD5E | BCD5 ON PS;
! Targ Sex x Part Age x Type of Agg (Hit v Rep outside fam) x Part Sex


! Five-Way Interactions

ABCD1E | ABCD1 ON PS;
! Targ Fam/NFam x Targ Sex x Part Age x Type of Agg (Hit v Yell) x Part Sex
ABCD2E | ABCD2 ON PS;
! Targ Fam/NFam x Targ Sex x Part Age x Type of Agg (Hit v Goss within fam) x Part Sex
ABCD3E | ABCD3 ON PS;
```

```
! Targ Fam/NFam x Targ Sex x Part Age x Type of Agg (Hit v Goss outside fam) x Part Sex
ABCD4E | ABCD4 ON PS;
! Targ Fam/NFam x Targ Sex x Part Age x Type of Agg (Hit v Rep within fam) x Part Sex
ABCD5E | ABCD5 ON PS;
! Targ Fam/NFam x Targ Sex x Part Age x Type of Agg (Hit v Rep outside fam) x Part Sex

%between CID%

E-ABCD5E@.01; Set random effect variances for E factors to facilitate estimation

OUTPUT: TECH8;

DEFINE:

! Recoded Main Effect Variables

! Needed to determine whether reporting and gossiping is within v outside fam
IF (WvO EQ 1) THEN WvO = 1; ! Within is 0, Outside is 1
IF (WvO EQ 2) THEN WvO = 0; ! Within is 0, Outside is 1

IF (Targ EQ 1) THEN TF = 0; ! Fam/NFam is 0, Non-Fam/NFam is 1
IF (Targ EQ 2) THEN TF = 1; ! Fam/NFam is 0, Non-Fam/NFam is 1
IF (Targ EQ 3) THEN TF = 1; ! Fam/NFam is 0, Non-Fam/NFam is 1
IF (Targ EQ 4) THEN TF = 1; ! Fam/NFam is 0, Non-Fam/NFam is 1
IF (Targ EQ 5) THEN TF = 1; ! Fam/NFam is 0, Non-Fam/NFam is 1
IF (Targ EQ 6) THEN TF = 0; ! Fam/NFam is 0, Non-Fam/NFam is 1

IF (Targ EQ 2) THEN TS = 0; ! Female is 0, Male is 1
IF (Targ EQ 3) THEN TS = 0; ! Female is 0, Male is 1
IF (Targ EQ 6) THEN TS = 0; ! Female is 0, Male is 1
IF (Targ EQ 1) THEN TS = 1; ! Female is 0, Male is 1
IF (Targ EQ 4) THEN TS = 1; ! Female is 0, Male is 1
IF (Targ EQ 5) THEN TS = 1; ! Female is 0, Male is 1

IF (AvC EQ 1) THEN CA = 1; ! Recode so Child is 0, Adult is 1
IF (AvC EQ 2) THEN CA = 0; ! Recode so Child is 0, Adult is 1

IF (AggAct EQ 2) THEN AT1 = 0; ! Hitting is referent
IF (AggAct EQ 2) THEN AT2 = 0; ! Hitting is referent
IF (AggAct EQ 2) THEN AT3 = 0; ! Hitting is referent
IF (AggAct EQ 2) THEN AT4 = 0; ! Hitting is referent
IF (AggAct EQ 2) THEN AT5 = 0; ! Hitting is referent

IF (AggAct EQ 4) THEN AT1 = 1; ! Yelling
IF (AggAct EQ 4) THEN AT2 = 0; ! Yelling
IF (AggAct EQ 4) THEN AT3 = 0; ! Yelling
IF (AggAct EQ 4) THEN AT4 = 0; ! Yelling
IF (AggAct EQ 4) THEN AT5 = 0; ! Yelling

IF (AggAct EQ 1 AND WvO EQ 0) THEN AT1 = 0; ! Gossiping within fam
IF (AggAct EQ 1 AND WvO EQ 0) THEN AT2 = 1; ! Gossiping within fam
IF (AggAct EQ 1 AND WvO EQ 0) THEN AT3 = 0; ! Gossiping within fam
IF (AggAct EQ 1 AND WvO EQ 0) THEN AT4 = 0; ! Gossiping within fam
IF (AggAct EQ 1 AND WvO EQ 0) THEN AT5 = 0; ! Gossiping within fam

IF (AggAct EQ 1 AND WvO EQ 1) THEN AT1 = 0; ! Gossiping outside fam
IF (AggAct EQ 1 AND WvO EQ 1) THEN AT2 = 0; ! Gossiping outside fam
IF (AggAct EQ 1 AND WvO EQ 1) THEN AT3 = 1; ! Gossiping outside fam
IF (AggAct EQ 1 AND WvO EQ 1) THEN AT4 = 0; ! Gossiping outside fam
IF (AggAct EQ 1 AND WvO EQ 1) THEN AT5 = 0; ! Gossiping outside fam

IF (AggAct EQ 3 AND WvO EQ 0) THEN AT1 = 0; ! Reporting within fam
IF (AggAct EQ 3 AND WvO EQ 0) THEN AT2 = 0; ! Reporting within fam
IF (AggAct EQ 3 AND WvO EQ 0) THEN AT3 = 0; ! Reporting within fam
IF (AggAct EQ 3 AND WvO EQ 0) THEN AT4 = 1; ! Reporting within fam
IF (AggAct EQ 3 AND WvO EQ 0) THEN AT5 = 0; ! Reporting within fam

IF (AggAct EQ 3 AND WvO EQ 1) THEN AT1 = 0; ! Reporting outside fam
IF (AggAct EQ 3 AND WvO EQ 1) THEN AT2 = 0; ! Reporting outside fam
IF (AggAct EQ 3 AND WvO EQ 1) THEN AT3 = 0; ! Reporting outside fam
IF (AggAct EQ 3 AND WvO EQ 1) THEN AT4 = 0; ! Reporting outside fam
```

```
IF (AggAct EQ 3 AND WvO EQ 1) THEN AT5 = 1; ! Reporting outside fam

! Calculated Two-Way Interaction Variables
TFTS  = TF*TS;
TFCA  = TF*CA;
TFAT1 = TF*AT1;
TFAT2 = TF*AT2;
TFAT3 = TF*AT3;
TFAT4 = TF*AT4;
TFAT5 = TF*AT5;
TSCA  = TS*CA;
TSAT1 = TS*AT1;
TSAT2 = TS*AT2;
TSAT3 = TS*AT3;
TSAT4 = TS*AT4;
TSAT5 = TS*AT5;
CAAT1 = CA*AT1;
CAAT2 = CA*AT2;
CAAT3 = CA*AT3;
CAAT4 = CA*AT4;
CAAT5 = CA*AT5;

! Calculated Three-Way Interaction Variables
TFTSCA = TF*TS*CA;  ! ABC
TFTSAT1 = TF*TS*AT1;! ABD1
TFTSAT2 = TF*TS*AT2;! ABD2
TFTSAT3 = TF*TS*AT3;! ABD3
TFTSAT4 = TF*TS*AT4;! ABD4
TFTSAT5 = TF*TS*AT5;! ABD5
TSCAAT1 = TS*CA*AT1;! ACD1
TSCAAT2 = TS*CA*AT2;! ACD2
TSCAAT3 = TS*CA*AT3;! ACD3
TSCAAT4 = TS*CA*AT4;! ACD4
TSCAAT5 = TS*CA*AT5;! ACD5
TFCAAT1 = TF*CA*AT1;! BCD1
TFCAAT2 = TF*CA*AT2;! BCD2
TFCAAT3 = TF*CA*AT3;! BCD3
TFCAAT4 = TF*CA*AT4;! BCD4
TFCAAT5 = TF*CA*AT5;! BCD5

! Calculated Four-Way Interaction Variable
TFTSCAA1 = TF*TS*CA*AT1;
TFTSCAA2 = TF*TS*CA*AT2;
TFTSCAA3 = TF*TS*CA*AT3;
TFTSCAA4 = TF*TS*CA*AT4;
TFTSCAA5 = TF*TS*CA*AT5;
```

MPLUS Output Table of Parameter Estimates

```
MODEL RESULTS

                                Posterior  One-Tailed         95% C.I.
                    Estimate       S.D.      P-Value   Lower 2.5%  Upper 2.5%
Significance

Within Level

 Residual Variances
    SCORE              0.326       0.001      0.000       0.324       0.329      *

Between IID Level

 Residual Variances
    SCORE              0.220       0.007      0.000       0.208       0.234      *
    A                  0.127       0.005      0.000       0.119       0.136      *
    B                  0.169       0.006      0.000       0.157       0.181      *
```

C	0.066	0.003	0.000	0.061	0.071	*
D1	0.167	0.007	0.000	0.154	0.180	*
D2	0.200	0.007	0.000	0.187	0.214	*
D3	0.185	0.007	0.000	0.172	0.200	*
D4	0.258	0.009	0.000	0.241	0.277	*
D5	0.120	0.005	0.000	0.111	0.130	*
AB	0.162	0.007	0.000	0.149	0.175	*
AC	0.020	0.002	0.000	0.016	0.024	*
AD1	0.180	0.008	0.000	0.165	0.197	*
AD2	0.071	0.005	0.000	0.061	0.082	*
AD3	0.119	0.006	0.000	0.106	0.131	*
AD4	0.213	0.009	0.000	0.195	0.232	*
AD5	0.062	0.005	0.000	0.053	0.072	*
BC	0.008	0.002	0.000	0.005	0.012	*
BD1	0.060	0.006	0.000	0.050	0.072	*
BD2	0.002	0.001	0.000	0.001	0.004	*
BD3	0.002	0.001	0.000	0.001	0.005	*
BD4	0.023	0.004	0.000	0.014	0.031	*
BD5	0.002	0.001	0.000	0.001	0.004	*
CD1	0.067	0.005	0.000	0.057	0.078	*
CD2	0.020	0.004	0.000	0.013	0.028	*
CD3	0.027	0.004	0.000	0.018	0.035	*
CD4	0.078	0.005	0.000	0.068	0.090	*
CD5	0.004	0.003	0.000	0.001	0.013	*
ABC	0.004	0.002	0.000	0.001	0.009	*
ABD1	0.059	0.007	0.000	0.045	0.073	*
ABD2	0.002	0.001	0.000	0.001	0.003	*
ABD3	0.002	0.001	0.000	0.001	0.004	*
ABD4	0.004	0.003	0.000	0.001	0.012	*
ABD5	0.002	0.002	0.000	0.001	0.007	*
ACD1	0.022	0.006	0.000	0.009	0.033	*
ACD2	0.002	0.001	0.000	0.001	0.006	*
ACD3	0.015	0.006	0.000	0.007	0.029	*
ACD4	0.046	0.006	0.000	0.034	0.059	*
ACD5	0.002	0.002	0.000	0.001	0.008	*
BCD1	0.009	0.006	0.000	0.003	0.024	*
BCD2	0.001	0.001	0.000	0.001	0.003	*
BCD3	0.001	0.001	0.000	0.001	0.004	*
BCD4	0.001	0.001	0.000	0.001	0.003	*
BCD5	0.001	0.000	0.000	0.001	0.003	*
ABCD1	0.003	0.003	0.000	0.001	0.011	*
ABCD2	0.001	0.001	0.000	0.001	0.004	*
ABCD3	0.002	0.001	0.000	0.001	0.005	*
ABCD4	0.002	0.000	0.000	0.001	0.003	*
ABCD5	0.001	0.001	0.000	0.001	0.004	*

Between CID Level

Means

SCORE	2.259	0.044	0.000	2.172	2.346	*
A	-0.929	0.039	0.000	-1.005	-0.852	*
B	0.016	0.026	0.276	-0.036	0.067	
C	-0.661	0.043	0.000	-0.745	-0.575	*
D1	0.835	0.052	0.000	0.732	0.940	*
D2	-0.633	0.056	0.000	-0.740	-0.519	*
D3	-0.741	0.053	0.000	-0.849	-0.639	*
D4	0.390	0.053	0.000	0.284	0.494	*
D5	-0.963	0.053	0.000	-1.064	-0.857	*
AB	0.109	0.030	0.000	0.052	0.169	*
AC	0.499	0.035	0.000	0.430	0.568	*
AD1	-0.206	0.043	0.000	-0.291	-0.120	*
AD2	0.987	0.054	0.000	0.881	1.094	*
AD3	1.181	0.056	0.000	1.071	1.293	*

AD4	0.266	0.045	0.000	0.178	0.356	*
AD5	1.069	0.048	0.000	0.975	1.164	*
BC	0.021	0.029	0.245	-0.035	0.077	
BD1	-0.051	0.031	0.046	-0.111	0.010	
BD2	-0.017	0.032	0.284	-0.082	0.044	
BD3	-0.054	0.035	0.058	-0.122	0.014	
BD4	-0.003	0.035	0.470	-0.071	0.065	
BD5	0.003	0.030	0.463	-0.054	0.062	
CD1	0.155	0.036	0.000	0.084	0.226	*
CD2	0.506	0.037	0.000	0.431	0.579	*
CD3	0.565	0.042	0.000	0.484	0.648	*
CD4	0.088	0.039	0.013	0.011	0.165	*
CD5	0.598	0.042	0.000	0.517	0.681	*
ABC	-0.047	0.033	0.080	-0.112	0.018	
ABD1	-0.073	0.036	0.026	-0.142	0.001	
ABD2	-0.235	0.035	0.000	-0.304	-0.165	*
ABD3	-0.231	0.036	0.000	-0.298	-0.160	*
ABD4	-0.300	0.040	0.000	-0.377	-0.223	*
ABD5	-0.090	0.035	0.002	-0.159	-0.024	*
ACD1	-0.286	0.038	0.000	-0.362	-0.212	*
ACD2	-0.465	0.038	0.000	-0.537	-0.390	*
ACD3	-0.566	0.040	0.000	-0.645	-0.487	*
ACD4	-0.216	0.037	0.000	-0.286	-0.142	*
ACD5	-0.596	0.039	0.000	-0.675	-0.520	*
BCD1	-0.069	0.041	0.055	-0.142	0.014	
BCD2	-0.036	0.039	0.186	-0.108	0.048	
BCD3	-0.018	0.039	0.318	-0.091	0.058	
BCD4	-0.003	0.042	0.466	-0.081	0.083	
BCD5	-0.028	0.039	0.252	-0.104	0.043	
ABCD1	0.181	0.047	0.000	0.080	0.267	*
ABCD2	0.078	0.046	0.057	-0.017	0.165	
ABCD3	0.145	0.044	0.001	0.056	0.227	*
ABCD4	0.090	0.048	0.029	-0.003	0.181	
ABCD5	0.048	0.046	0.150	-0.033	0.138	
E	-0.463	0.036	0.000	-0.534	-0.391	*
AE	0.338	0.037	0.000	0.267	0.409	*
BE	0.599	0.043	0.000	0.516	0.684	*
CE	0.232	0.041	0.000	0.152	0.313	*
DE1	0.091	0.044	0.019	0.005	0.176	*
DE2	0.349	0.043	0.000	0.261	0.430	*
DE3	0.360	0.043	0.000	0.274	0.444	*
DE4	0.102	0.043	0.010	0.017	0.186	*
DE5	0.431	0.043	0.000	0.342	0.512	*
ABE	0.034	0.048	0.243	-0.062	0.125	
ACE	-0.140	0.045	0.000	-0.230	-0.051	*
AD1E	-0.176	0.049	0.000	-0.268	-0.075	*
AD2E	-0.467	0.045	0.000	-0.554	-0.380	*
AD3E	-0.415	0.047	0.000	-0.508	-0.319	*
AD4E	-0.420	0.049	0.000	-0.516	-0.324	*
AD5E	-0.363	0.047	0.000	-0.451	-0.268	*
BCE	-0.180	0.056	0.000	-0.294	-0.075	*
BD1E	-0.279	0.056	0.000	-0.387	-0.171	*
BD2E	-0.479	0.052	0.000	-0.579	-0.377	*
BD3E	-0.513	0.052	0.000	-0.614	-0.411	*
BD4E	-0.394	0.054	0.000	-0.501	-0.290	*
BD5E	-0.559	0.056	0.000	-0.668	-0.450	*
CD1E	-0.243	0.055	0.000	-0.346	-0.130	*
CD2E	-0.204	0.053	0.000	-0.301	-0.095	*
CD3E	-0.214	0.052	0.000	-0.312	-0.111	*
CD4E	-0.193	0.053	0.000	-0.300	-0.091	*
CD5E	-0.226	0.055	0.000	-0.333	-0.122	*
ABCE	-0.091	0.063	0.085	-0.209	0.039	
ABD1E	0.306	0.064	0.000	0.180	0.435	*

ABD2E	0.151	0.060	0.005	0.038	0.273	*
ABD3E	0.196	0.060	0.001	0.078	0.313	*
ABD4E	0.189	0.060	0.000	0.077	0.309	*
ABD5E	0.056	0.064	0.192	-0.068	0.185	
ACD1E	0.255	0.063	0.000	0.125	0.372	*
ACD2E	0.194	0.062	0.001	0.070	0.309	*
ACD3E	0.198	0.061	0.001	0.072	0.311	*
ACD4E	0.246	0.060	0.000	0.128	0.367	*
ACD5E	0.190	0.062	0.000	0.073	0.312	*
BCD1E	0.245	0.079	0.000	0.091	0.399	*
BCD2E	0.141	0.075	0.033	-0.008	0.284	
BCD3E	0.202	0.072	0.002	0.063	0.346	*
BCD4E	0.149	0.074	0.017	0.009	0.296	*
BCD5E	0.135	0.078	0.037	-0.011	0.295	
ABCD1E	-0.166	0.093	0.037	-0.356	0.018	
ABCD2E	0.031	0.088	0.349	-0.138	0.205	
ABCD3E	-0.065	0.083	0.208	-0.229	0.104	
ABCD4E	-0.062	0.084	0.231	-0.230	0.096	
ABCD5E	0.038	0.091	0.342	-0.151	0.207	
Variances						
SCORE	0.039	0.015	0.000	0.020	0.079	*
A	0.026	0.011	0.000	0.014	0.056	*
B	0.004	0.003	0.000	0.001	0.012	*
C	0.034	0.014	0.000	0.018	0.071	*
D1	0.051	0.021	0.000	0.027	0.106	*
D2	0.060	0.023	0.000	0.032	0.121	*
D3	0.050	0.020	0.000	0.027	0.104	*
D4	0.053	0.022	0.000	0.028	0.113	*
D5	0.052	0.021	0.000	0.028	0.109	*
AB	0.007	0.004	0.000	0.003	0.018	*
AC	0.016	0.007	0.000	0.007	0.034	*
AD1	0.028	0.012	0.000	0.014	0.061	*
AD2	0.052	0.021	0.000	0.028	0.106	*
AD3	0.057	0.023	0.000	0.031	0.118	*
AD4	0.032	0.014	0.000	0.016	0.070	*
AD5	0.038	0.016	0.000	0.019	0.080	*
BC	0.002	0.001	0.000	0.001	0.005	*
BD1	0.002	0.002	0.000	0.001	0.008	*
BD2	0.006	0.003	0.000	0.002	0.015	*
BD3	0.009	0.005	0.000	0.004	0.022	*
BD4	0.009	0.005	0.000	0.003	0.021	*
BD5	0.003	0.002	0.000	0.001	0.008	*
CD1	0.011	0.006	0.000	0.004	0.028	*
CD2	0.013	0.007	0.000	0.005	0.033	*
CD3	0.023	0.011	0.000	0.010	0.052	*
CD4	0.016	0.008	0.000	0.007	0.037	*
CD5	0.023	0.010	0.000	0.011	0.051	*
ABC	0.002	0.001	0.000	0.001	0.006	*
ABD1	0.004	0.003	0.000	0.001	0.013	*
ABD2	0.006	0.004	0.000	0.002	0.016	*
ABD3	0.005	0.004	0.000	0.001	0.014	*
ABD4	0.012	0.007	0.000	0.004	0.030	*
ABD5	0.003	0.002	0.000	0.001	0.009	*
ACD1	0.008	0.005	0.000	0.002	0.023	*
ACD2	0.009	0.006	0.000	0.002	0.025	*
ACD3	0.014	0.008	0.000	0.005	0.035	*
ACD4	0.007	0.005	0.000	0.002	0.021	*
ACD5	0.012	0.007	0.000	0.004	0.030	*
BCD1	0.002	0.002	0.000	0.001	0.007	*
BCD2	0.002	0.001	0.000	0.001	0.006	*
BCD3	0.002	0.001	0.000	0.001	0.005	*
BCD4	0.002	0.002	0.000	0.001	0.009	*

BCD5	0.001	0.001	0.000	0.001	0.005	*
ABCD1	0.003	0.002	0.000	0.001	0.010	*
ABCD2	0.002	0.002	0.000	0.001	0.009	*
ABCD3	0.002	0.002	0.000	0.001	0.009	*
ABCD4	0.003	0.003	0.000	0.001	0.011	*
ABCD5	0.002	0.002	0.000	0.001	0.008	*
E	0.010	0.000	0.000	0.010	0.010	
AE	0.010	0.000	0.000	0.010	0.010	
BE	0.010	0.000	0.000	0.010	0.010	
CE	0.010	0.000	0.000	0.010	0.010	
DE1	0.010	0.000	0.000	0.010	0.010	
DE2	0.010	0.000	0.000	0.010	0.010	
DE3	0.010	0.000	0.000	0.010	0.010	
DE4	0.010	0.000	0.000	0.010	0.010	
DE5	0.010	0.000	0.000	0.010	0.010	
ABE	0.010	0.000	0.000	0.010	0.010	
ACE	0.010	0.000	0.000	0.010	0.010	
AD1E	0.010	0.000	0.000	0.010	0.010	
AD2E	0.010	0.000	0.000	0.010	0.010	
AD3E	0.010	0.000	0.000	0.010	0.010	
AD4E	0.010	0.000	0.000	0.010	0.010	
AD5E	0.010	0.000	0.000	0.010	0.010	
BCE	0.010	0.000	0.000	0.010	0.010	
BD1E	0.010	0.000	0.000	0.010	0.010	
BD2E	0.010	0.000	0.000	0.010	0.010	
BD3E	0.010	0.000	0.000	0.010	0.010	
BD4E	0.010	0.000	0.000	0.010	0.010	
BD5E	0.010	0.000	0.000	0.010	0.010	
CD1E	0.010	0.000	0.000	0.010	0.010	
CD2E	0.010	0.000	0.000	0.010	0.010	
CD3E	0.010	0.000	0.000	0.010	0.010	
CD4E	0.010	0.000	0.000	0.010	0.010	
CD5E	0.010	0.000	0.000	0.010	0.010	
ABCE	0.010	0.000	0.000	0.010	0.010	
ABD1E	0.010	0.000	0.000	0.010	0.010	
ABD2E	0.010	0.000	0.000	0.010	0.010	
ABD3E	0.010	0.000	0.000	0.010	0.010	
ABD4E	0.010	0.000	0.000	0.010	0.010	
ABD5E	0.010	0.000	0.000	0.010	0.010	
ACD1E	0.010	0.000	0.000	0.010	0.010	
ACD2E	0.010	0.000	0.000	0.010	0.010	
ACD3E	0.010	0.000	0.000	0.010	0.010	
ACD4E	0.010	0.000	0.000	0.010	0.010	
ACD5E	0.010	0.000	0.000	0.010	0.010	
BCD1E	0.010	0.000	0.000	0.010	0.010	
BCD2E	0.010	0.000	0.000	0.010	0.010	
BCD3E	0.010	0.000	0.000	0.010	0.010	
BCD4E	0.010	0.000	0.000	0.010	0.010	
BCD5E	0.010	0.000	0.000	0.010	0.010	
ABCD1E	0.010	0.000	0.000	0.010	0.010	
ABCD2E	0.010	0.000	0.000	0.010	0.010	
ABCD3E	0.010	0.000	0.000	0.010	0.010	
ABCD4E	0.010	0.000	0.000	0.010	0.010	

Note that although the five-way interaction was not statistically significant (i.e., none of the between-country [Between CID level] means for the variables reflecting the 5-way interaction were significant), all but one of the four-way interactions were significant; the exception being the ABCE interaction term reflecting family status x target sex x participant age x participant sex, but not involving type of aggression. In the text, we discuss the pattern of results for four of these five factors (i.e., type of aggression, family status, participant age, and

participant sex) but do not incorporate the fifth factor of target sex, as it was not a part of our hypotheses and the effects in this model remain consistent with our primary hypotheses. Nevertheless, we provide a summary here of the patterns involving target sex: for direct aggression, female and male participants exhibited similarly high levels of aggression toward their sisters and brothers, except when males aggressed toward their sisters (i.e., they exhibited less aggression toward sisters). For friends/acquaintances, female and male participants exhibited similarly high levels of aggression toward males and females, except when males aggressed toward other males (i.e., they exhibited more aggression toward other males).

For indirect aggression—gossiping and reporting outside of the family—there were relatively low levels of aggression and only small differences that did not reflect a consistent pattern. For reporting within the family, however, a pattern similar to direct aggression was observed when aggressing toward family members. When aggressing toward friends/acquaintances, females aggressing toward their female friends/acquaintances were the most aggressive, with all other combinations reflecting similar and lower levels of aggression.

Appendix F: List of international collaborators by country with email contact information

Country	Name	Email
Turkey	Suzan Ceylan-Batur	scbatur@etu.edu.tr
Italy	Silvia Galdi	silvia.galdi@unicampania.it
Italy	Chiara Ambrosio	chiara.ambrosio@unicampania.it
France	Dimitri Dubois	dimitri.dubois@umontpellier.fr
France/Sweden	Arnaud Tognetti	arnaud.tognetti@umontpellier.fr
Sweden	Anja Winter	anja.winter@ki.se
Sweden	Evelina Thunell	evelina.thunell@ki.se
Chile	Ana María Fernandez	ana.fernandez@usach.cl
Chile	Maria Teresa Barbato	maria.barbato@usach.cl
Romania	Oana David	oana.david@ubbcluj.ro
Romania	Ioana Iuga	ioana.iuga@ubbcluj.ro
Sweden	Eric Skoog	erisko@prio.org
Canada	Daniel Sznycer	daniel.sznycer@okstate.edu
Canada	Yunsuh Nike Wee	yunsuh.wee@okstate.edu
Canada	Igor Grossmann	igrossma@uwaterloo.ca
Canada	Torin Peter Young	tpyoung@uwaterloo.ca
Pakistan	Muhammad Rizwan	muhammad.rizwan@numspak.edu.pk
Germany	Timur Sevincer	*timur.sevincer@leuphana.de*
Germany	Anna Ziska	*anna.ziska@yahoo.de*
Slovakia	Michal Kohút	michal.kohut@truni.sk
Slovakia	Peter Halama	peter.halama@savba.sk
Lebanon	Laith Al-Shawaf	lalshawa@uccs.edu
Brazil	Anthonieta Looman Mafra	looman.anthonieta@gmail.com
Brazil	Jaroslava Varella Valentova	jarkavalentova@gmail.com
Brazil	Marco Antonio Correa Varella	macvarella@gmail.com
Colombia	Oscar Javier Galindo Caballero	ojgalindo@ucatolica.edu.co
Colombia	David Guzman	davidl.guzmang@konradlorenz.edu.co
Colombia	Danilo Zambrano	danilo.zambranor@konradlorenz.edu.co
Colombia	Lady Javela	lady.javela@urosario.edu.co
Colombia	Julio Eduardo Cruz	jecruz@uniandes.edu.co
Australia	Takeshi Hamamura	takeshi.hamamura@curtin.edu.au
UK	Ayse K. Uskul	a.k.uskul@sussex.ac.uk
Senegal	Oumar Barry	oumar.barry@ucad.edu.sn
Senegal	Laina Ngom Dieng	laina.ngom@ucad.edu.sn
Senegal	Dieyanaba Gabrielle Ndiaye	dieynabagabrielle.ndiaye@ucad.edu.sn
New Zealand	Johannes A. Karl	johannes.karl@vuw.ac.nz
Spain	Beatriz Perez Sánchez	perezbeatriz@uniovi.es
UK	Jiaqing O	jiaqingo@um.edu.mo

**Austria**	**Eduard Brandstätter**	**eduard.brandstaetter@jku.at**
**South Korea**	**Jaewuk Jung**	**jeiwuk@sogang.ac.kr**
**Thailand**	**Watcharaporn Boonyasiriwat**	**watcharaporn.p@chula.ac.th**
**Czechia**	**Sylvie Graf**	**sylvie.graf@psu.cas.cz**
**Czechia**	**Martina Hřebíčková**	**martina@psu.cas.cz**
**South Korea**	**Jinseok P. Kim**	**pjkim@yonsei.ac.kr**
**South Korea**	**Eunkook M. Suh**	**esuh@yonsei.ac.kr**
**Bolivia**	**Eric Roth Unzueta**	**eroth@ucb.edu.bo**

Appendix G: Details of GDP, GII, and Cultural Distance variables

The variables Gross Domestic Product (GDP), Gender Inequality Index (GII), and Cultural Distance were all drawn from the Ecology-Culture Dataset (1), a resource for understanding cultural variation.

GDP is a way to measure the monetary value of goods and services produced within a country. It is used to assess a country's overall economic development and wealth.

GII measures gender inequality in reproductive health, empowerment (such as education or political representation), and economic participation. Higher values indicate greater gender inequality.

Cultural Distance (2) measures cultural dissimilarity between countries based on World Values Survey responses, with higher values indicating greater cultural differences, usually compared to the United States.

References

1. Wormley, A. S., Kwon, J. Y., Barlev, M., & Varnum, M. E. (2022). The Ecology-Culture Dataset: A new resource for investigating cultural variation. *Scientific Data*, *9*(1), 615.

2. Muthukrishna, M., Bell, A. V., Henrich, J., Curtin, C. M., Gedranovich, A., McInerney, J., & Thue, B. (2020). Beyond Western, Educated, Industrial, Rich, and Democratic (WEIRD) psychology: Measuring and mapping scales of cultural and psychological distance. *Psychological science*, *31*(6), 678-701.

Questionnaire

Kin Aggression PSY 350 2024

Start of Block: Informed Consent

Q1 **Past Behaviors and Relationship Inferences Study**

I am a graduate student under the direction of Professor Doug Kenrick in the Department of Psychology at Arizona State University. I am inviting your participation, which will involve answering questions about your life experiences and inferences. You must be 18 years or older to participate. You have the right not to answer any question, and to stop participation at any time. Although there is no direct benefit to you other than learning about how research is conducted, possible benefits of your participation are helping researchers gain a better understanding behaviors in different types of relationships. There are no foreseeable risks or discomforts to your participation. No identifying personal information connected to your data will be collected in order to protect your confidentiality. All of your data will remain confidential and secure. Your responses will be anonymous. The results of this study may be used in reports, presentations, or publications but your name will not be used.

If you have any questions concerning the research study, please contact Amanda Kirsch at apk@asu.edu or Dr. Doug Kenrick at douglas.kenrick@asu.edu. If you have any questions about your rights as a participant in this research, or if you feel you have been placed at risk, you can contact the Chair of the Human Subjects Institutional Review Board, through the ASU Office of Research Integrity and Assurance, at (480) 965-6788. By pressing the button below you are agreeing to be a part of the study.

○ I agree (1)

End of Block: Informed Consent

Start of Block: sib q

Q19 Do you have at least one biological sibling (that you share both parents with)?

○ Yes (1)

○ No (2)

End of Block: sib q

Start of Block: sorry

Q96 Sorry, unfortunately you are not eligible to participate in this study.

End of Block: sorry

Start of Block: childhood

Q2 **For the following questions, imagine behaviors you did ONLY during your childhood or early adolescence (up until the age of 16).**

Q3 Have you ever hit/slapped **a sister**?

- Never (1)
- Once (2)
- Several times (3)
- Many times (4)
- I don't have a sister (5)

Q4 Have you ever hit/slapped **a brother**?

- Never (1)
- Once (2)
- Several times (3)
- Many times (4)
- I don't have a brother (5)

Q5 Have you ever hit/slapped **a male friend**?

- Never (1)
- Once (2)
- Several times (3)
- Many times (4)
- I did not have any male friends (5)

Q6 Have you ever hit/slapped **a female friend**?

- Never (1)
- Once (2)
- Several times (3)
- Many times (4)
- I did not have any female friends (5)

Q7 Have you ever hit/slapped **a male acquaintance**?

- Never (1)
- Once (2)
- Several times (3)
- Many times (4)

Q11 Have you ever hit/slapped **a female acquaintance**?

- Never (1)
- Once (2)
- Several times (3)
- Many times (4)

Q8 Have you ever yelled at **a sister**?

- Never (1)
- Once (2)
- Several times (3)
- Many times (4)
- I don't have a sister (5)

Q9 Have you ever yelled at **a brother**?

- Never (1)
- Once (2)
- Several times (3)
- Many times (4)
- I don't have a brother (5)

Q12 Have you ever yelled at **a male friend**?

- Never (1)
- Once (2)
- Several times (3)
- Many times (4)
- I didn't have any male friends (5)

Q13 Have you ever yelled at **a female friend**?

- Never (1)
- Once (2)
- Several times (3)
- Many times (4)
- I didn't have any female friends (5)

Q14 Have you ever yelled at **a male acquaintance**?

- Never (1)
- Once (2)
- Several times (3)
- Many times (4)

Q15 Have you ever yelled at **a female acquaintance**?

- Never (1)
- Once (2)
- Several times (3)
- Many times (4)

Q16 Have you ever reported **a sister to one of your parents** after they did something bad?

- Never (1)
- Once (2)
- Several times (3)
- Many times (4)
- I don't have a sister (5)

Q17 Have you ever reported **a brother to one of your parents** after they did something bad?

- Never (1)
- Once (2)
- Several times (3)
- Many times (4)
- I don't have a brother (5)

Q18 Have you ever reported **a sister to an authority figure (outside of your family)** after they did something bad?

- Never (1)
- Once (2)
- Several times (3)
- Many times (4)
- I don't have a sister (5)

Q19 Have you ever reported **a brother to an authority figure (outside of your family)** after they did something bad?

- Never (1)
- Once (2)
- Several times (3)
- Many times (4)
- I don't have a brother (5)

Q20 Have you ever reported **a male friend to one of your parents** after they did something bad?

- Never (1)
- Once (2)
- Several times (3)
- Many times (4)
- I didn't have any male friends (5)

Q21 Have you ever reported **a female friend to one of your parents** after they did something bad?

- Never (1)
- Once (2)
- Several times (3)
- Many times (4)
- I didn't have any female friends (5)

Q85 This is an attention check. Please select the answer "Once"

- Never (1)
- Once (2)
- Several times (3)
- Many times (4)

Q22 Have you ever reported **a male friend to an authority figure (outside of your family)** after they did something bad?

- Never (1)
- Once (2)
- Several times (3)
- Many times (4)
- I didn't have any male friends (5)

Q23 Have you ever reported **a female friend to an authority figure (outside of your family)** after they did something bad?

- Never (1)
- Once (2)
- Several times (3)
- Many times (4)
- I didn't have any female friends (5)

Q24 Have you ever reported **a male acquaintance to one of your parents** after they did something bad?

- Never (1)
- Once (2)
- Several times (3)
- Many times (4)

Q25 Have you ever reported **a female acquaintance to one of your parents** after they did something bad?

- Never (1)
- Once (2)
- Several times (3)
- Many times (4)

Q26 Have you ever reported **a male acquaintance to an authority figure (outside of your family)** after they did something bad?

- Never (1)
- Once (2)
- Several times (3)
- Many times (4)

Q27 Have you ever reported **a female acquaintance to an authority figure (outside of your family)** after they did something bad?

- Never (1)
- Once (2)
- Several times (3)
- Many times (4)

Q28 Have you ever talked about **a sister** behind her back **to another member of your family** in a way that was intended to damage her reputation or exclude her from a group?

- Never (1)
- Once (2)
- Several times (3)
- Many times (4)
- I don't have a sister (5)

Q29 Have you ever talked about **a brother** behind his back **to another member of your family** in a way that was intended to damage his reputation or exclude him from a group?

- Never (1)
- Once (2)
- Several times (3)
- Many times (4)
- I don't have a brother (5)

Q30 Have you ever talked about **a sister** behind her back **to someone outside of your family** in a way that was intended to damage her reputation or exclude her from a group?

- Never (1)
- Once (2)
- Several times (3)
- Many times (4)
- I don't have a sister (5)

Q31 Have you ever talked about **a brother** behind his back **to someone outside of your family** in a way that was intended to damage his reputation or exclude him from a group?

- Never (1)
- Once (2)
- Several times (3)
- Many times (4)
- I don't have a brother (5)

Q32 Have you ever talked about **a male friend** behind his back **to another member of your family** in a way that was intended to damage his reputation or exclude him from a group?

- Never (1)
- Once (2)
- Several times (3)
- Many times (4)
- I didn't have any male friends (5)

Q33 Have you ever talked about **a male friend** behind his back **to someone outside of your family** in a way that was intended to damage his reputation or exclude him from a group?

- Never (1)
- Once (2)
- Several times (3)
- Many times (4)
- I didn't have any male friends (5)

Q34 Have you ever talked about **a female friend** behind her back **to another member of your family** in a way that was intended to damage her reputation or exclude her from a group?

- Never (1)
- Once (2)
- Several times (3)
- Many times (4)
- I didn't have any female friends (5)

Q35 Have you ever talked about **a female friend** behind her back **to someone outside of your family** in a way that was intended to damage her reputation or exclude her from a group?

- Never (1)
- Once (2)
- Several times (3)
- Many times (4)
- I didn't have any female friends (5)

Q36 Have you ever talked about **a male acquaintance** behind his back **to another member of your family** in a way that was intended to damage his reputation or exclude him from a group?

- Never (1)
- Once (2)
- Several times (3)
- Many times (4)

Q37 Have you ever talked about **a male acquaintance** behind his back **to someone outside of your family** in a way that was intended to damage his reputation or exclude him from a group?

- Never (1)
- Once (2)
- Several times (3)
- Many times (4)

Q38 Have you ever talked about **a female acquaintance** behind her back **to another member of your family** in a way that was intended to damage her reputation or exclude her from a group?

- Never (1)
- Once (2)
- Several times (3)
- Many times (4)

Q86 This is an attention check. Please select the answer "Never"

- Never (1)
- Once (2)
- Several times (3)
- Many times (4)

Q39 Have you ever talked about **a female acquaintance** behind her back **to someone outside of your family** in a way that was intended to damage her reputation or exclude her from a group?

- Never (1)
- Once (2)
- Several times (3)
- Many times (4)

End of Block: childhood

Start of Block: Adulthood

Q40 **For the following questions, imagine behaviors you did ONLY as an adult (18 years old or older).**

Q41 Have you ever hit/slapped **a sister**?

- Never (1)
- Once (2)
- Several times (3)
- Many times (4)
- I don't have a sister (5)

Q42 Have you ever hit/slapped **a brother**?

- Never (1)
- Once (2)
- Several times (3)
- Many times (4)
- I don't have a brother (5)

Q43 Have you ever hit/slapped **a male friend**?

- Never (1)
- Once (2)
- Several times (3)
- Many times (4)
- I do not have any male friends (5)

Q44 Have you ever hit/slapped **a female friend**?

- Never (1)
- Once (2)
- Several times (3)
- Many times (4)
- I do not have any female friends (5)

Q45 Have you ever hit/slapped **a male acquaintance**?

- Never (1)
- Once (2)
- Several times (3)
- Many times (4)

Q46 Have you ever hit/slapped **a female acquaintance**?

- Never (1)
- Once (2)
- Several times (3)
- Many times (4)

Q47 Have you ever yelled at **a sister**?

- Never (1)
- Once (2)
- Several times (3)
- Many times (4)
- I don't have a sister (5)

Q48 Have you ever yelled at **a brother**?

- Never (1)
- Once (2)
- Several times (3)
- Many times (4)
- I don't have a brother (5)

Q49 Have you ever yelled at **a male friend**?

- Never (1)
- Once (2)
- Several times (3)
- Many times (4)
- I do not have any male friends (5)

Q50 Have you ever yelled at **a female friend**?

- Never (1)
- Once (2)
- Several times (3)
- Many times (4)
- I do not have any female friends (5)

Q51 Have you ever yelled at **a male acquaintance**?

- Never (1)
- Once (2)
- Several times (3)
- Many times (4)

Q52 Have you ever yelled at **a female acquaintance**?

- Never (1)
- Once (2)
- Several times (3)
- Many times (4)

Q87 This is an attention check. Please select the option "Many times"

- Never (1)
- Once (2)
- Several times (3)
- Many times (4)

Q53 Have you ever reported **a sister to one of your parents** after they did something bad?

- Never (1)
- Once (2)
- Several times (3)
- Many times (4)
- I don't have a sister (5)

Q54 Have you ever reported **a brother to one of your parents** after they did something bad?

- Never (1)
- Once (2)
- Several times (3)
- Many times (4)
- I don't have a brother (5)

Q55 Have you ever reported **a sister to an authority figure (outside of your family)** after they did something bad?

- Never (1)
- Once (2)
- Several times (3)
- Many times (4)
- I don't have a sister (5)

Q56 Have you ever reported **a brother to an authority figure (outside of your family)** after they did something bad?

- Never (1)
- Once (2)
- Several times (3)
- Many times (4)
- I don't have a brother (5)

Q57 Have you ever reported **a male friend to one of your parents** after they did something bad?

- Never (1)
- Once (2)
- Several times (3)
- Many times (4)
- I don't have any male friends (5)

Q58 Have you ever reported **a female friend to one of your parents** after they did something bad?

- Never (1)
- Once (2)
- Several times (3)
- Many times (4)
- I don't have any female friends (5)

Q59 Have you ever reported **a male friend to an authority figure (outside of your family)** after they did something bad?

- Never (1)
- Once (2)
- Several times (3)
- Many times (4)
- I don't have any male friends (5)

Q60 Have you ever reported **a female friend to an authority figure (outside of your family)** after they did something bad?

- Never (1)
- Once (2)
- Several times (3)
- Many times (4)
- I don't have any female friends (5)

Q61 Have you ever reported **a male acquaintance to one of your parents** after they did something bad?

- Never (1)
- Once (2)
- Several times (3)
- Many times (4)

Q62 Have you ever reported **a female acquaintance to one of your parents** after they did something bad?

- Never (1)
- Once (2)
- Several times (3)
- Many times (4)

Q63 Have you ever reported **a male acquaintance to an authority figure (outside of your family)** after they did something bad?

- Never (1)
- Once (2)
- Several times (3)
- Many times (4)

Q64 Have you ever reported **a female acquaintance to an authority figure (outside of your family)** after they did something bad?

- Never (1)
- Once (2)
- Several times (3)
- Many times (4)

Q65 Have you ever talked about **a sister** behind her back **to another member of your family** in a way that was intended to damage her reputation or exclude her from a group?

- Never (1)
- Once (2)
- Several times (3)
- Many times (4)
- I don't have a sister (5)

Q66 Have you ever talked about **a brother** behind his back **to another member of your family** in a way that was intended to damage his reputation or exclude him from a group?

- Never (1)
- Once (2)
- Several times (3)
- Many times (4)
- I don't have a brother (5)

Q67 Have you ever talked about **a sister** behind her back **to someone outside of your family** in a way that was intended to damage her reputation or exclude her from a group?

- Never (1)
- Once (2)
- Several times (3)
- Many times (4)
- I don't have a sister (5)

Q68 Have you ever talked about **a brother** behind his back **to someone outside of your family** in a way that was intended to damage his reputation or exclude him from a group?

- Never (1)
- Once (2)
- Several times (3)
- Many times (4)
- I don't have a brother (5)

Q69 Have you ever talked about **a male friend** behind his back **to another member of your family** in a way that was intended to damage his reputation or exclude him from a group?

- Never (1)
- Once (2)
- Several times (3)
- Many times (4)
- I don't have any male friends (5)

Q88 This is an attention check. Please select the option "Several times"

- Never (1)
- Once (2)
- Several times (3)
- Many times (4)

Q70 Have you ever talked about **a male friend** behind his back **to someone outside of your family** in a way that was intended to damage his reputation or exclude him from a group?

- Never (1)
- Once (2)
- Several times (3)
- Many times (4)
- I don't have any male friends (5)

Q71 Have you ever talked about **a female friend** behind her back **to another member of your family** in a way that was intended to damage her reputation or exclude her from a group?

- Never (1)
- Once (2)
- Several times (3)
- Many times (4)
- I don't have any female friends (5)

Q72 Have you ever talked about **a female friend** behind her back **to someone outside of your family** in a way that was intended to damage her reputation or exclude her from a group?

- Never (1)
- Once (2)
- Several times (3)
- Many times (4)
- I don't have any female friends (5)

Q73 Have you ever talked about **a male acquaintance** behind his back **to another member of your family** in a way that was intended to damage his reputation or exclude him from a group?

- Never (1)
- Once (2)
- Several times (3)
- Many times (4)

Q74 Have you ever talked about **a male acquaintance** behind his back **to someone outside of your family** in a way that was intended to damage his reputation or exclude him from a group?

- Never (1)
- Once (2)
- Several times (3)
- Many times (4)

Q75 Have you ever talked about **a female acquaintance** behind her back **to another member of your family** in a way that was intended to damage her reputation or exclude her from a group?

- Never (1)
- Once (2)
- Several times (3)
- Many times (4)

Q76 Have you ever talked about **a female acquaintance** behind her back **to someone outside of your family** in a way that was intended to damage her reputation or exclude her from a group?

- Never (1)
- Once (2)
- Several times (3)
- Many times (4)

End of Block: Adulthood

Start of Block: sister

Q12 Please carefully read each statement and indicate how morally wrong each statement is, from 1 (morally acceptable) to 5 (extremely immoral).

	Morally acceptable 1 (1)	2 (2)	3 (3)	4 (4)	Extremely immoral 5 (5)
Intentionally slapping a sister (1)	○	○	○	○	○
Intentionally hitting a sister with a fist (2)	○	○	○	○	○
Yelling at a sister (3)	○	○	○	○	○
Verbally assaulting a sister (4)	○	○	○	○	○
Spreading harmful gossip about a sister behind her back (5)	○	○	○	○	○
Sharing an embarrassing story about a sister that she would not want to be shared (6)	○	○	○	○	○
Calling the police on a sister for stealing money from her company (7)	○	○	○	○	○
Contacting a sister's school about suspected cheating on an exam (10)	○	○	○	○	○

Killing a sister (12)	○	○	○	○	○

End of Block: sister

Start of Block: Block 7

Q98 Click to write the question text

○ Click to write Choice 1 (1)

○ Click to write Choice 2 (2)

○ Click to write Choice 3 (3)

Q2 Please carefully read each statement and indicate how morally wrong each statement is, from 1 (morally acceptable) to 5 (extremely immoral).

	Morally acceptable 1 (1)	2 (2)	3 (3)	4 (4)	Extremely immoral 5 (5)
Intentionally slapping a brother (1)	○	○	○	○	○
Intentionally hitting a brother with a fist (2)	○	○	○	○	○
Yelling at a brother (3)	○	○	○	○	○
Verbally assaulting a brother (4)	○	○	○	○	○
Spreading harmful gossip about a brother behind his back (5)	○	○	○	○	○
Sharing an embarrassing story about a brother that he would not want to be shared (6)	○	○	○	○	○
Calling the police on a brother for stealing money from his company (7)	○	○	○	○	○
Contacting a brother's school about suspected cheating on an exam (8)	○	○	○	○	○

Killing a brother (9)	○	○	○	○	○

End of Block: Block 7

Start of Block: Block 8

Q8 Please carefully read each statement and indicate how morally wrong each statement is, from 1 (morally acceptable) to 5 (extremely immoral).

	Morally acceptable 1 (1)	2 (2)	3 (3)	4 (4)	Extremely immoral 5 (5)
Intentionally slapping a female acquaintance (1)					
Intentionally hitting a female acquaintance with a fist (2)					
Yelling at a female acquaintance (3)					
Verbally assaulting a female acquaintance (4)					
Spreading harmful gossip about a female acquaintance behind her back (5)					
Sharing an embarrassing story about a female acquaintance that she would not want to be shared (6)					

Calling the police on a female acquaintance for stealing money from hercompany (7)	○	○	○	○	○
Contacting a female acquaintance's school about suspected cheating on an exam (8)	○	○	○	○	○
Killing a female acquaintance (9)	○	○	○	○	○

End of Block: Block 8

Start of Block: Block 9

Q14 Please carefully read each statement and indicate how morally wrong each statement is, from 1 (morally acceptable) to 5 (extremely immoral).

	Morally acceptable 1 (1)	2 (2)	3 (3)	4 (4)	Extremley immoral 5 (5)
Intentionally slapping a female friend (1)	o	o	o	o	o
Intentionally hitting a female friend with a fist (2)	o	o	o	o	o
Yelling at a female friend (3)	o	o	o	o	o
Verbally assaulting a female friend (4)	o	o	o	o	o
Spreading harmful gossip about a female friend behind her back (5)	o	o	o	o	o
Sharing an embarrassing story about a female friend that she would not want to be shared (6)	o	o	o	o	o
Calling the police on a female friend for stealing money from her company (7)	o	o	o	o	o

Contacting a female friend's school about suspected cheating on an exam (8)	○	○	○	○	○
Killing a female friend (9)	○	○	○	○	○

End of Block: Block 9

Start of Block: Block 10

Q9 Please carefully read each statement and indicate how morally wrong each statement is, from 1 (morally acceptable) to 5 (extremely immoral).

	Morally acceptable 1 (1)	2 (2)	3 (3)	4 (4)	Extremely immoral 5 (5)
Intentionally slapping a male friend (1)	○	○	○	○	○
Intentionally hitting a male friend with a fist (2)	○	○	○	○	○
Yelling at a male friend (3)	○	○	○	○	○
Verbally assaulting a male friend (4)	○	○	○	○	○
Spreading harmful gossip about a male friend behind his back (5)	○	○	○	○	○
Sharing an embarrassing story about a male friend that he would not want to be shared (6)	○	○	○	○	○
Calling the police on a male friend for stealing money from his company (7)	○	○	○	○	○

Contacting a male friend's school about suspected cheating on an exam (8)	○	○	○	○	○
Killing a male friend (9)	○	○	○	○	○

End of Block: Block 10

Start of Block: Block 11

Q13 Please carefully read each statement and indicate how morally wrong each statement is, from 1 (morally acceptable) to 5 (extremely immoral).

	Morally acceptable 1 (1)	2 (2)	3 (3)	4 (4)	Extremely immoral 5 (5)
Intentionally slapping a male acquaintance (1)	○	○	○	○	○
Intentionally hitting a male acquaintance with a fist (2)	○	○	○	○	○
Yelling at a male acquaintance (3)	○	○	○	○	○
Verbally assaulting a male acquaintance (4)	○	○	○	○	○
Spreading harmful gossip about a male acquaintance behind his back (5)	○	○	○	○	○
Sharing an embarrassing story about a male acquaintance that he would not want to be shared (6)	○	○	○	○	○

Calling the police on a male acquaintance for stealing money from his company (7)	○	○	○	○	○
Contacting a male acquaintance's school about suspected cheating on an exam (8)	○	○	○	○	○
Killing a male acquaintance (9)	○	○	○	○	○

End of Block: Block 11

Start of Block: Demographics

Q77 How many full biological siblings do you have?

▼ 1 (1) ... 10 or more (10)

Q78 What is your sex?

○ Male (1)

○ Female (2)

○ Other (3) ____

Q83 What is your age?

▼ 18 (1) ... 102 (85)

Q99 What is your race/ethnicity? Please select what you most identify with.

- ○ White (10)
- ○ Black (11)
- ○ Native American (12)
- ○ Asian (13)
- ○ Native Hawaiian or Pacific Islander (14)
- ○ Middle Eastern (15)
- ○ More than one race (16)
- ○ Other (17) ____

End of Block: Demographics

Start of Block: ladder

Q81

Q82 Think of this ladder as where people stand in The United States. At the **top** of the ladder are the people who are best off- those who have the most money, the most education and the most respected jobs. At the **bottom** are the people who are the worst off- who have the least money, least education, and the least respected jobs or no job. The higher up you are on this ladder, the closer you are to the people at the very top; the lower you are, the closer you are to the people at the very bottom.

Where would you place yourself on this ladder?

	1	2	3	4	5	6	7	8	9	10
Please slide the bar to the number where you would place yourself on the ladder ()										

End of Block: ladder

Start of Block: Debriefing

Q84 PLEASE READ THIS DEBRIEFING. THEN CLICK THE BUTTON AT THE BOTTOM TO FINISH THE STUDY. Thank you for participating in this study. Your participation has helped contribute to an important cross-cultural research study. Now that we are done with the study, I'd like to tell you a little bit more about what we were investigating.
In this study we were exploring how common aggressive behaviors are across different relationship types. You were asked how many times you have performed an aggressive action against different individuals. We are particularly interested in forms of aggression that take place with biological relatives.
This study was conducted across numerous different countries around the world. Once we have finished data collection, our team plans to analyze the data, write it up in a research article, and publish the results. Thank you so much for your participation, and for contributing to psychological research.
If you have any questions concerning the research study, please contact Amanda Kirsch at apkirsc1@asu.edu.

End of Block: Debriefing
